# CD45-mediated control of TCR tuning in naïve and memory CD8^+^ T cells

**DOI:** 10.1038/ncomms13373

**Published:** 2016-11-14

**Authors:** Jae-Ho Cho, Hee-Ok Kim, Young-Jun Ju, Yoon-Chul Kye, Gil-Woo Lee, Sung-Woo Lee, Cheol-Heui Yun, Nunzio Bottini, Kylie Webster, Christopher C. Goodnow, Charles D. Surh, Cecile King, Jonathan Sprent

**Affiliations:** 1 Academy of Immunology and Microbiology, Institute for Basic Science, Pohang 790-784, Korea; 2 Division of Integrative Biosciences and Biotechnology, Pohang University of Science and Technology, Pohang 790-784, Korea; 3 Immunology Research Program, Garvan Institute of Medical Research, Darlinghurst, New South Wales 2010, Australia; 4 Department of Agricultural Biotechnology, Seoul National University, Seoul 151-921, Korea; 5 La Jolla Institute for Allergy and Immunology, La Jolla, California 92037, USA

## Abstract

Continuous contact with self-major histocompatibility complex (MHC) ligands is essential for survival of naïve T cells but not memory cells. This surprising finding implies that T cell subsets may vary in their relative T-cell receptor (TCR) sensitivity. Here we show that in CD8^+^T cells TCR sensitivity correlates inversely with levels of CD5, a marker for strong self-MHC reactivity. We also show that TCR sensitivity is lower in memory CD8^+^ T cells than naïve cells. In both situations, TCR hypo-responsiveness applies only to short-term TCR signalling events and not to proliferation, and correlates directly with increased expression of a phosphatase, CD45 and reciprocal decreased expression of activated LCK. Inhibition by high CD45 on CD8^+^ T cells may protect against overt TCR auto-MHC reactivity, while enhanced sensitivity to cytokines ensures strong responses to foreign antigens.

## Abstract (web summary)

Naïve T cells establish self-tolerance via negative selection of cells with strong reactivity for self-peptide/MHC complexes, but undergo T-cell receptor (TCR) desensitisation when leaving the thymus. Here, Cho *et al.* show that TCR desensitisation correlates with cell-surface density of the phosphatase CD45.

Differentiation of naïve T cells into memory cells leads to enhanced responses to foreign antigens with retention of tolerance to self-antigens^1^,^2^. For naïve T cells, self-tolerance is established in the thymus through negative selection of cells with strong reactivity for self-peptide/major histocompatibility complexes (MHCs) (self-pMHC) plus positive selection of cells with low but significant affinity for self-pMHC (ref. 3). Especially for CD8^+^ cells, naïve T-cell recognition of self-pMHC ligands in the extra-thymic environment is essential for cell survival: such recognition elicits low-level TCR signals which, together with IL-7, upregulate Bcl-2 and promote long-term survival of naïve CD8^+^ T cells in interphase^4^,^5^.

Since naïve T cells undergoing positive selection in the thymus are presumed to vary in their degree of self-pMHC reactivity, cells with the highest affinity (just below the level leading to negative selection) would be potentially dangerous in the post-thymic environment. Because of this problem, positively selected T cells are subjected to a process of mild TCR desensitisation before leaving the thymus^6^,^7^. Such TCR “tuning” occurs during the differentiation of mature CD4^+^ and CD8^+^ single-positive (SP) cells from CD4^+^CD8^+^ double-positive (DP) precursors and is associated with upregulation of negative regulators of TCR signalling, notably CD5, and downregulation of microRNA (miR)-181a which inhibits expression of negative regulatory protein tyrosine phosphatases (PTPs)^8^,^9^. Although TCR tuning is presumed to reduce reactivity to self-pMHC ligands and thereby promote self-tolerance, direct support for this notion is sparse. The relevant question here is whether mature T cells with high innate self-pMHC reactivity, for example, naïve T cells with high expression of CD5 (CD5^hi^ cells), show lower TCR sensitivity than CD5^lo^ cells. In fact, there is evidence against this idea. Thus, for naïve T cells, CD5^hi^ cells show higher background expression of tyrosine-phosphorylated CD3ζ than CD5^lo^ cells^10^,^11^. Also, CD5^hi^ cells display stronger lymphopenia-driven homeostatic proliferation (HP) as well as antigen-specific expansion than CD5^lo^ cells^10^,^12^,^13^. These findings are not easy to reconcile with the notion that self-reactivity is regulated by TCR tuning, at least as defined by relative CD5 expression.

Although continuous contact with self-pMHC ligands is essential for naïve CD8^+^ T cells, memory CD8^+^ T cells can survive in the absence of these ligands^14^. This finding is surprising because the enhanced expression of adhesion molecules on memory cells would be expected to augment contact with self-pMHC, especially on antigen-presenting cells (APC). One explanation for this finding is that differentiation of naïve into memory CD8^+^ T cells reduces their TCR sensitivity. This idea seems unlikely because memory CD8^+^ T cells generally give enhanced proliferative responses to antigen^15^,^16^,^17^. However, this is not invariably the case. Thus, as defined by phosphorylation (p) of ERK after contact with specific antigen, TCR sensitivity of naïve and memory CD8^+^ T cells was reported to be indistinguishable^18^. Moreover, a recent study found reduced TCR sensitivity of memory CD8^+^ T cells relative to naïve cells for p-ZAP-70 induction^19^. Like two others^20^,^21^, this study also reported that memory CD8^+^ T cells gave lower proliferative responses to antigen than naïve CD8^+^ T cells. By contrast, many other studies found that memory CD8^+^ T cells gave better proliferative response than naïve cells^15^,^16^,^22^.

In this paper, we sought evidence of TCR tuning in mature CD8^+^ T cells by multiple parameters, first in CD5^lo^ versus CD5^hi^ subsets of naïve cells, and then in naïve versus memory cells. For naïve CD8^+^ T cells, the results show that CD5^hi^ cells are less TCR sensitive than CD5^lo^ cells but are more sensitive to cytokines. Likewise, memory CD8^+^ T cells have lower TCR sensitivity than naïve cells but increased sensitivity to cytokines, accounting for their increased responsiveness to antigen. In each situation, TCR sensitivity correlates inversely with cell-surface density of CD45.

## Results

### Proliferation versus TCR signalling in naïve CD8^+^ T-cell subsets

**Figure 1:**
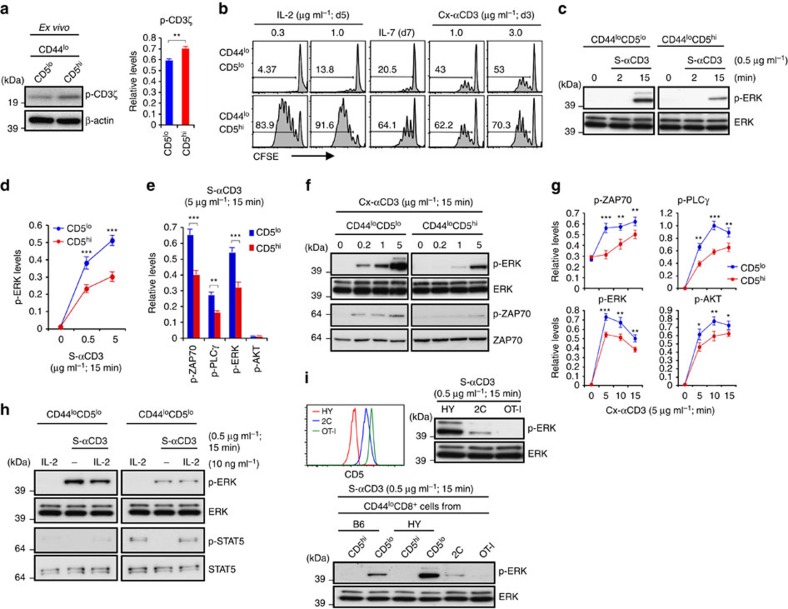
TCR sensitivity of CD5^lo^ versus CD5^hi^ naïve CD8^+^ T cells. (**a**) Levels of tyrosine-phosphorylated CD3ζ chain (p-CD3ζ; 21 kDa) in freshly isolated CD5^lo^ and CD5^hi^ B6 naïve (CD44^lo^) CD8^+^ T cells (relative to β-actin; mean±s.d.). (**b**) Proliferation of CFSE-labelled CD5^lo^ and CD5^hi^ B6 naïve CD8^+^ T cells after incubation with IL-2, IL-7, or cross-linked anti-CD3 (Cx-αCD3) mAb. (**c**–**e**) ERK phosphorylation (**c**) and densitometric levels (relative to total ERK; **d** or β-actin; **e**) of phosphorylated ERK (**d**,**e**), ZAP-70 (**e**), PLCγ (**e**), and AKT (**e**) in CD5^lo^ and CD5^hi^ B6 naïve CD8^+^ T cells after incubation with soluble anti-CD3 (S-αCD3) mAb (graphs in **d** and **e** show mean±s.d.). (**f**,**g**) Phosphorylation of ERK and ZAP70 (**f**) and levels of phosphorylated ZAP-70, PLCγ, ERK and AKT (relative to β-actin; mean±s.d.) (**g**) in CD5^lo^ and CD5^hi^ B6 naïve CD8^+^ T cells after incubation with Cx-αCD3 mAb. (**h**) Phosphorylation of ERK and STAT5 in CD5^lo^ and CD5^hi^ B6 naïve CD8^+^ T cells after S-αCD3 mAb incubation with or without IL-2. (**i**) CD5 levels by flow cytometry (top left) and S-αCD3 mAb-induced ERK phosphorylation by Western blot (top right) in naïve CD8^+^ T cells from HY, 2C and OT-I TCR Tg mice (top and bottom) or CD5^lo^ and CD5^hi^ naïve CD8^+^ T cells from B6 and HY mice (bottom); note that CD5^hi^ HY cells are TCR-clonotype negative. Data are representative of at least four (**a**,**c**-**g**) and three independent experiments (**b**,**h**,**i**). Unpaired Student's *t*-test was used for the statistical analysis. *P<0.05; **P<0.005; ***P<0.0005.

In initial experiments, FACS-sorted CD5^lo^ and CD5^hi^ subsets of naïve CD44^lo^ CD8^+^ T cells (Supplementary Fig. 1a) were analysed for expression of tyrosine-phosphorylated CD3ζ (p-CD3ζ). In agreement with prior studies on naïve T cells^10^,^11^, CD5^hi^ CD44^lo^ CD8^+^ T cells prepared from young C57BL/6 (B6) mice showed higher background expression of p-CD3ζ than CD5^lo^ cells (Fig. 1a). Likewise, as for HP in lymphopenic hosts^12^, CD5^hi^ cells displayed more extensive proliferation (CFSE dilution) than CD5^lo^ cells when cultured with cross-linked anti-CD3 monoclonal antibody (Cx-αCD3 mAb) *in vitro* (Fig. 1b). Collectively, these data would seem to argue against the concept of TCR tuning of mature T cells. However, for resting T cells, phosphorylation of CD3ζ is only partial and is therefore not necessarily indicative of TCR signalling^23^. Also, as shown elsewhere^12^, stronger proliferation of CD5^hi^ than CD5^lo^ CD8^+^ T cells following CD3 ligation may simply reflect the enhanced sensitivity of CD5^hi^ cells to cytokines, including endogenous IL-2 (Fig. 1b). Indeed, CD3 mAb-induced proliferation of both subsets was minimal following IL-2 blockade or with IL-2-deficient naïve CD8^+^ T cells (Supplementary Fig. 1b,c).

To examine the influence of CD5 expression on downstream TCR signalling, purified naïve (CD44^lo^) populations of CD5^hi^ and CD5^lo^ CD8^+^ T cells were cultured with soluble anti-CD3 (S-αCD3) mAb to examine phosphorylation of ERK (p-ERK) and other signalling molecules by immunoblot analysis. Here, the striking finding was that induction of p-ERK, p-PLCγ and p-ZAP-70 after brief (2–15 min) exposure to S-αCD3 mAb was clearly more prominent in CD5^lo^ cells than in CD5^hi^ cells (Fig. 1c–e). Similar findings applied following culture with strongly stimulatory Cx-αCD3 mAb (Fig. 1f,g and Supplementary Fig. 1d); note that, unlike Cx-αCD3, culture with S-αCD3 failed to cause T-cell activation (Supplementary Fig. 1e) and is therefore more relevant to the weak TCR signalling induced when CD8^+^ T cells encounter self-pMHC ligands under *in vivo* conditions.

The poor induction of p-ERK by CD5^hi^ CD8^+^ T cells after CD3 ligation was not seen following culture with phorbol myristate acetate (PMA)±ionomycin (Supplementary Fig. 1f), indicating no intrinsic signalling defect, and could not be overcome by addition of IL-2, despite strong induction of p-STAT5 in these cells (Fig. 1h). As for polyclonal CD8^+^ T cells, the inverse correlation between CD5 expression and p-ERK induction applied to TCR transgenic (Tg) mice. Thus, for CD8^+^ T cells from TCR Tg lines with high CD5 expression, namely OT-1 and 2C, these cells showed much weaker p-ERK induction after CD3 ligation than CD8^+^ T cells from the HY line where the clonotype-positive cells are all CD5^lo^ cells (Fig. 1i, upper); by contrast, the minor component of HY CD5^hi^ cells with endogenous TCR showed much weaker p-ERK (Fig. 1i, lower). Similar findings were applied to CD69 upregulation. Thus, as for p-ERK induction, HY CD8^+^ T cells showed much higher CD69 upregulation at 3 h after CD3 ligation than OT-1 CD8^+^ T cells (Supplementary Fig. 1g); note that, relative to p-ERK induction, CD69 induction was slow, being very low at 1 h.

The above findings indicated that, in marked contrast to p-CD3ζ expression and proliferative responses, naïve CD5^hi^ CD8^+^ T cells showed weaker downstream TCR signalling than CD5^lo^ cells. As discussed later, CD5^hi^ naïve CD8^+^ T cells also showed a reduced Ca^2+^ flux following CD3 ligation and, notably, reduced constitutive expression of p-LCK relative to CD5^lo^ cells (see below).

### TCR sensitivity of naïve versus memory CD8^+^ T cells

The implication from the above data is that naïve T cells with “above-average” affinity for self-components undergo a form of TCR desensitisation that prevents the cells from displaying overt auto-reactivity; perhaps because these cells have enhanced sensitivity to IL-2, the cells do not show diminished responses to foreign antigens but rather exhibit enhanced responses^10^,^13^. What then happens to TCR reactivity when naïve T cells switch to memory cells?

**Figure 2:**
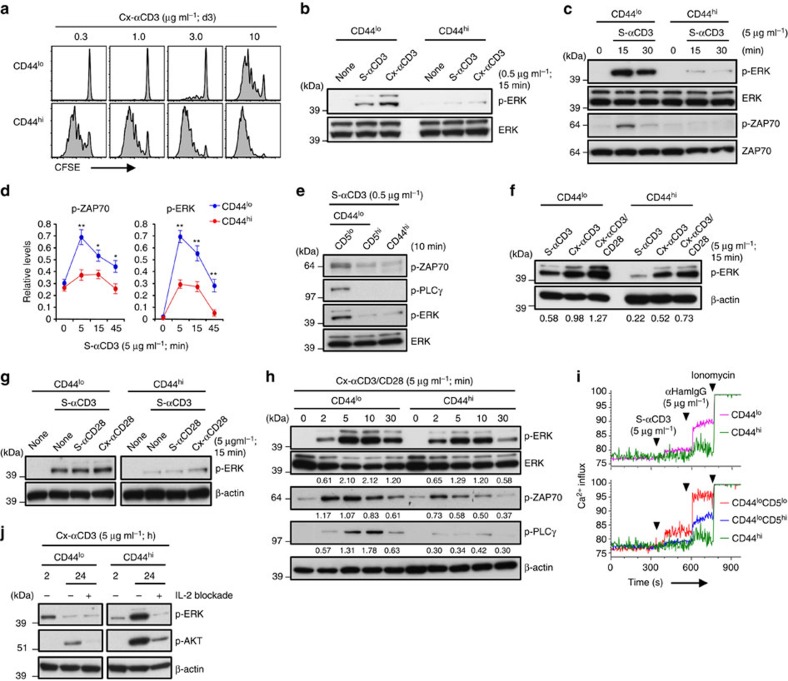
TCR sensitivity of naïve versus memory CD8^+^ T cells. (**a**) Proliferation of CFSE-labelled CD44^lo^ naïve and CD44^hi^ memory-phenotype (MP) B6 CD8^+^ T cells after incubation with Cx-αCD3 mAb. (**b**) ERK phosphorylation in CD44^lo^ and CD44^hi^ B6 CD8^+^ T cells after incubation with S-αCD3 or Cx-αCD3 mAb. (**c**,**d**) Phosphorylation of ERK and ZAP70 (**c**) and densitometric levels (relative to β-actin; mean±s.d.) (**d**) in CD44^lo^ and CD44^hi^ B6 CD8^+^ T cells after incubation with S-αCD3 mAb. (**e**) Phosphorylation of ERK, ZAP-70, and PLCγ in CD44^lo^ CD5^lo^ and CD5^hi^ and CD44^hi^ B6 CD8^+^ T cells after incubation with S-αCD3 mAb. (**f**,**g**) ERK phosphorylation in CD44^lo^ and CD44^hi^ B6 CD8^+^ T cells after incubation with S- or Cx-αCD3±Cx-αCD28 (**f**) and S-αCD3±S- or Cx-αCD28 mAbs (**g**). (**h**) Phosphorylation of ERK, ZAP-70, and PLCγ in CD44^lo^ and CD44^hi^ CD8^+^ T cells after incubation with Cx-αCD3 and -αCD28 mAbs. (**i**) Flow cytometry for Ca^2+^ flux in CD44^lo^ and CD44^hi^ (top) or CD44^lo^ CD5^lo^ and CD5^hi^ and CD44^hi^ CD8^+^ T cells (bottom) gated from Indo-1-loaded total B6 LN cells after incubation with the indicated stimuli. (**j**) Phosphorylation of ERK and AKT in CD44^lo^ and CD44^hi^ B6 CD8^+^ T cells at 2 and 24 h after incubation with Cx-αCD3 mAb±αIL-2 mAb blockade. Data are representative of three (**a**,**b**,**e**–**j**) and at least four independent experiments (**c**,**d**). Unpaired Student's *t*-test was used for the statistical analysis. *P<0.005; **P<0.0005.

As mentioned earlier, memory T cells generally give stronger and more rapid responses to foreign antigens than naïve T cells^15^,^16^,^22^. In support of this dogma, CD8^+^ T cells with a memory (CD44^hi^) phenotype (MP) prepared from B6 mice (Supplementary Fig. 2a) gave far stronger proliferative responses (CFSE dilution) to Cx-αCD3 than naïve CD44^lo^ CD8^+^ T cells (Fig. 2a). However, CD44^hi^ MP cells are known to secrete more IL-2 (Supplementary Fig. 2b) and display a higher density of IL-2Rβ (CD122) than naïve cells (Supplementary Fig. 2c). Hence, better responses of MP cells to antigen could reflect heightened cytokine responsiveness to endogenous IL-2 (Supplementary Fig. 2d). If so, the relevant question is whether MP cells show altered early TCR responsiveness. Here, the striking finding was that, following culture with either S-αCD3 (Fig. 2b–g) or Cx-αCD3 (Fig. 2b,f,h), MP CD44^hi^ CD8^+^ T cells showed much weaker p-ERK and also p-ZAP-70 induction than naïve CD44^lo^ CD8^+^ T cells; for p-ERK, weaker induction in CD44^hi^ than CD44^lo^ cells also applied for flow cytometry via intracellular staining with p-ERK mAb (Supplementary Fig. 2e–g) and was in accord with decreased upregulation of CD69 expression in CD44^hi^ cells (Supplementary Fig. 2h). Likewise, induction of a Ca^2+^ flux after exposure to Cx-αCD3 was far lower for CD44^hi^ CD8^+^ T cells than for CD44^lo^ cells (Fig. 2i, upper); for the latter, consistent with the above data on p-ERK induction (Fig. 1c–h), CD44^lo^ CD5^lo^ cells showed higher Ca^2+^ fluxes than CD5^hi^ cells (Fig. 2i, lower and Supplementary Fig. 2i). Consistent with the role of PLCγ in Ca^2+^ signalling^24^, the limited Ca^2+^ flux seen with CD3 ligation of CD44^hi^ MP cells correlated with reduced p-PLCγ in these cells relative to CD44^lo^ naïve cells (Fig. 2e,h).

For the mitogen-activated protein kinase pathways, weak induction of p-ERK by CD3 ligation in CD44^hi^ cells could not be overcome by addition of costimulation via CD28 ligation (Fig. 2f,g) and did not apply to p-p38 or p-JNK induction (Supplementary Fig. 2j). For p-ERK induction, weaker induction in CD44^hi^ MP cells than CD44^lo^ naïve CD8^+^ T cells was apparent from 5 min to 2 h after CD3 stimulation. Surprisingly, this difference was reversed at 24 h: at this time, p-ERK induction in CD44^hi^ MP cells rose to very high levels and was paralleled by strong induction of p-AKT (Fig. 2j). Notably, such signalling was abolished by mAb blockade of IL-2, indicating that the strong induction of p-ERK and p-AKT in the cultures at 24 h reflected rapid production of IL-2 by the memory cells.

### TCR sensitivity of antigen-induced memory CD8^+^ T cells

**Figure 3:**
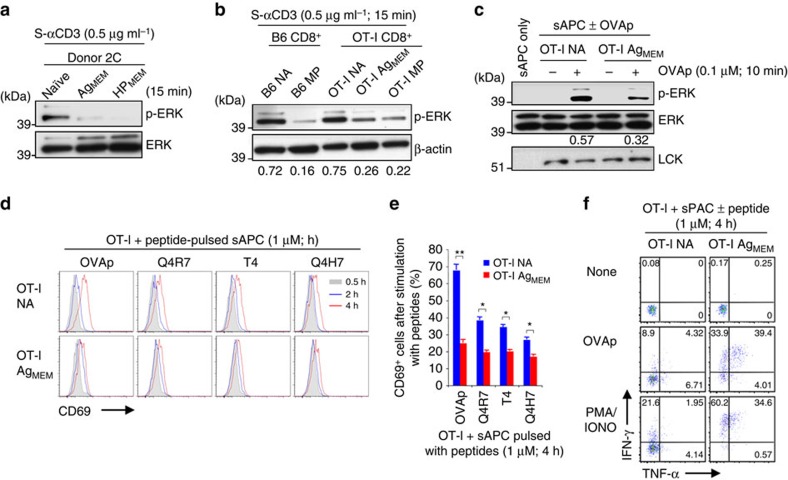
TCR desensitisation in induced memory CD8^+^ T cells. (**a**) Naïve 2C CD8^+^ T cells were transferred into irradiated B6 mice and then immunized with specific peptide (Ag_MEM_; *n*=5) or left unimmunized to form memory cells via lymphopenia-induced proliferation (HP_MEM_; *n*=4). The memory 2C cells purified at 56 d and freshly isolated naïve 2C cells (*n*=3) as controls were analysed for ERK phosphorylation after incubation with S-αCD3 mAb. (**b**) Memory OT-I CD8^+^ T cells obtained by adoptive transfer into B6 hosts and immunization with OVAp-pulsed BMDC (OT-I Ag_MEM_; *n*=5), together with host-derived B6 naïve (NA) CD44^lo^ and CD44^hi^ (MP) CD8^+^ T cells, were analysed for ERK phosphorylation after incubation with S-αCD3 mAb. Naïve and MP OT-I cells from unimmunized OT-I Tg mice (*n*=3) were used as control. (**c**) OT-I naïve and Ag_MEM_ obtained as in **b** were incubated with either unpulsed or OVAp-pulsed splenic APC (sAPC) and analysed for ERK phosphorylation; note that LCK is not detectable with sAPC only. (**d**,**e**) Flow cytometry for CD69 upregulation (**d**) and percentages of CD69^+^ cells (**e**; mean±s.d.) on OT-I naïve and memory cells as in **c** after incubation with sAPC pulsed with OVAp and its variant peptides, Q4R7, T4 and Q4H7. (**f**) IFN-γ and TNF-α production from OT-I naïve and memory cells as in **c** after incubation with sAPC±OVAp or PMA plus ionomycin (PMA/IONO). Data are representative of two (**f**) and three (**a**-**e**) independent experiments. Unpaired Student's *t*-test was used for the statistical analysis. *P<0.005; **P<0.0005.

Since MP cells in normal mice are not necessarily the counterpart of memory cells primed to defined antigens, we examined the TCR sensitivity of antigen-induced memory CD8^+^ T cells. As for the polyclonal CD44^hi^ cells from B6 mice used above, poor p-ERK induction following short-term CD3 ligation also applied to experimentally induced memory CD8^+^ T cells, namely naïve (NA) CD44^lo^ 2C TCR Tg CD8^+^ T cells primed with specific immunogenic peptide *in vivo* (Ag_MEM_) and also to 2C cells converted from naïve CD44^lo^ to memory CD44^hi^ cells as the result of HP in lymphopenic hosts (HP_MEM_) (Fig. 3a and Supplementary Fig. 3a). Similar findings applied to OT-1 TCR Tg CD8^+^ T cells; here, lower p-ERK induction in memory cells than naïve cells applied to Ag_MEM_ OT-1 cells (Fig. 3b and Supplementary Fig 3b,c) and also to the naturally occurring subset of CD44^hi^ MP OT-1 cells present in unimmunized OT-I mice (Fig. 3b and Supplementary Fig. 3b). Thus, for each type of memory cells examined, p-ERK induction was lower than in naïve cells (Supplementary Fig. 3d).

The above data refer to TCR signalling induced by CD3 ligation. Essentially similar findings applied following contact with specific antigenic peptide presented by APC. Thus, when OT-1 CD8^+^ T cells were stimulated with APC pulsed with ovalbumin peptide 257–264 (OVAp), p-ERK induction was lower in Ag_MEM_ cells than in naïve cells (Fig. 3c). Similar findings applied to upregulation of the activation marker CD69, both for strong peptide (OVAp) and weaker peptides (Q4R7, T4, Q4H7) (Fig. 3d,e); likewise, OT-1 Ag_MEM_ cells were less sensitive to downregulation of CD62L than naïve cells, especially with weaker peptides at early time points (30 min) (Supplementary Fig. 3e). However, in marked contrast to these early TCR signalling events, cytokine synthesis by OT-I Ag_MEM_ cells, like B6 MP cells, was invariably far stronger than for naïve cells (Fig. 3f and Supplementary Fig. 3f,g).

Collectively, these findings indicated that both MP and induced memory CD8^+^ T cells were less sensitive to TCR ligation than naïve cells, thus paralleling the data on naïve CD5^hi^ versus CD5^lo^ subsets.

### Cause of TCR desensitisation in memory CD8^+^ T cells

**Figure 4:**
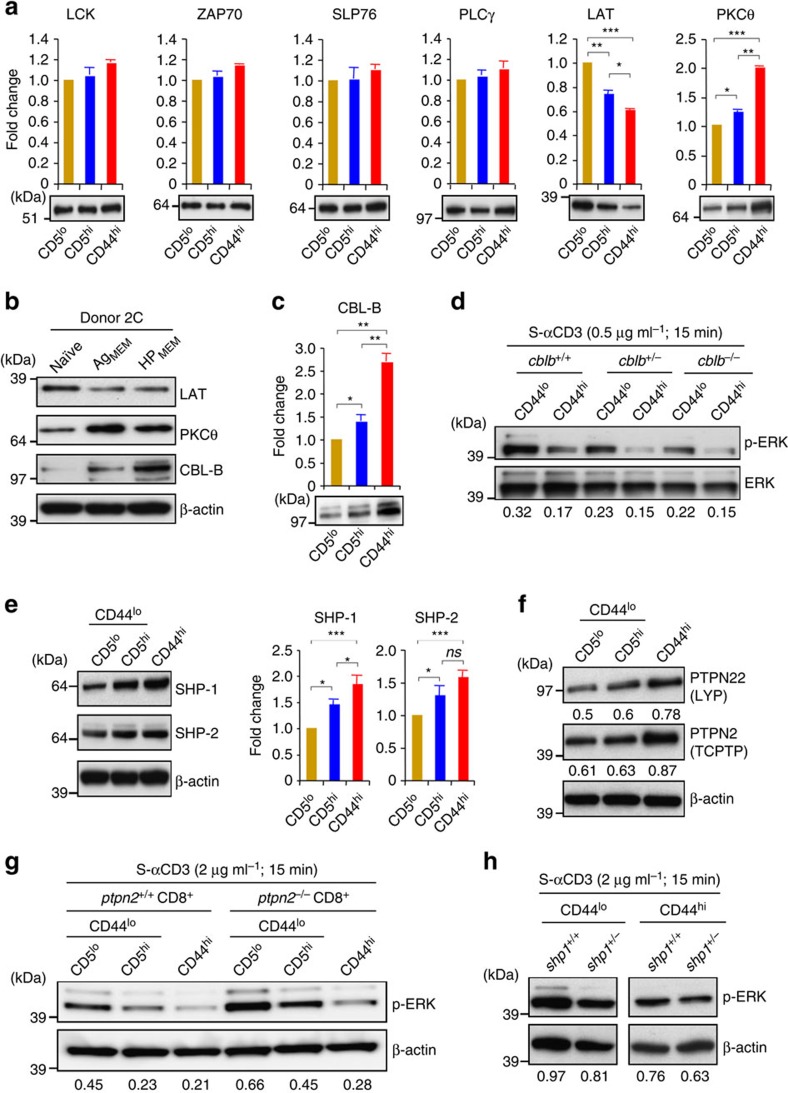
Expression of TCR signalling proteins in CD8^+^ T cell subsets. (**a**) Expression levels of TCR signalling complex proteins, LCK, ZAP-70, SLP-76, PLCγ, LAT and PKCθ in CD44^lo^ B6 naïve CD5^lo^ and CD5^hi^ and CD44^hi^ MP CD8^+^ T cells, shown as fold change of densitometric levels (relative to β-actin; mean±s.d.). (**b,c**) Expression levels of LAT, PKCθ and CBL-B in 2C naïve, Ag_MEM_ and HP_MEM_ CD8^+^ T cells as in Fig. 3a (**b**) and CBL-B levels in B6 CD44^lo^ CD5^lo^ and CD5^hi^ and CD44^hi^ CD8^+^ T cells (**c**; mean±s.d.). (**d**) ERK phosphorylation in CD44^lo^ and CD44^hi^ CD8^+^ T cells from *cblb*^*+/+*^, *cblb*^*+/−*^ or *cblb*^*−/−*^ mice after incubation with S-αCD3 mAb. (**e**) Expression (Western blot and densitometry) of SHP-1 and SHP-2 in CD44^lo^ B6 naïve CD5^lo^ and CD5^hi^ and CD44^hi^ MP CD8^+^ T cells (mean±s.d.). (**f**) Expression of PTPN22 (LYP) and PTPN2 (TCPTP) in CD44^lo^ B6 naïve CD5^lo^ and CD5^hi^ and CD44^hi^ MP CD8^+^ T cells. (**g**) ERK phosphorylation in CD44^lo^ naïve CD5^lo^ and CD5^hi^ cells and CD44^hi^ MP CD8^+^ T cells from *ptpn2*^*+/+*^ (*Ptpn2*^*fl/fl*^) and *ptpn2*^*−/−*^ (*Lck-cre.Ptpn2*^*fl/fl*^) mice after incubation with S-αCD3 mAb. (**h**) ERK phosphorylation in CD44^lo^ and CD44^hi^ CD8^+^ T cells from *shp1*^*+/+*^ and *shp1*^*+/−*^ mice (derived from SHP-1-deficient *me*^*v*^*/me*^*v*^ mice) after incubation with S-αCD3 mAb. Data are representative of three (**a**–**e**) and two independent experiments (**f**–**g**). Unpaired Student's *t*-test was used for the statistical analysis. *P<0.05; **P<0.005; ***P<0.0005; *ns,* not significant.

CD44^lo^ naïve and CD44^hi^ MP CD8^+^ T cells displayed equivalent expression of total LCK, ZAP-70, SLP-76 and PLCγ but did show altered expression of LAT and PKCθ (Fig. 4a). Thus, LAT was highest in CD5^lo^ naïve cells, intermediate in CD5^hi^ naïve cells and lowest in MP cells, whereas the reverse applied to levels of PKCθ. Similar findings applied to both 2C naïve and memory cells, with notable parallel increase of CBL-B, a negative regulator of TCR signalling^25^ (Fig. 4b). Hence, TCR sensitivity correlated directly with total LAT expression but inversely with CBL-B expression (Fig. 4b,c). This finding raised the question whether the reduced TCR sensitivity of MP cells reflected enhanced expression of CBL-B. This possibility seems unlikely, however, because MP CD8^+^ T cells prepared from *cblb*^*−/−*^ mice did not show improved p-ERK induction after CD3 ligation relative to *cblb*^*+/+*^ or *cblb*^*+/−*^ cells (Fig. 4d). An obvious question is whether memory cells show enhanced expression of CD5, a negative regulator for TCR signalling. This appeared to be the case for B6 MP cells as reported previously^12^ but, both for Ag_MEM_ and HP_MEM_ cells, CD5 levels were lower in these induced memory cells than in naïve cells (Supplementary Fig. 4a and see below).

We then explored the possibility that memory cells show increased expression of other negative regulators of TCR signalling, namely certain PTPs. In support of this idea, relative to naïve cells, CD44^hi^ MP CD8^+^ T cells displayed a striking reduction in miR-181a, a known regulator of a number of PTPs (ref. 9), and increased mRNA expression of DUSP5, though not several other PTPs (Supplementary Fig. 4b). At the protein level, however, MP cells showed increased expression of SHP-1 and SHP-2 (ref. 26) (Fig. 4e), and two recently reported negative regulators PTPN2 (TCPTP) and PTPN22 (LYP)^27^,^28^ (Fig. 4f). If these or other PTPs (ref. 29) accounted for the weak TCR sensitivity of memory cells, then adding PTP inhibitors would be expected to enhance TCR signalling. However, tests on five different PTP inhibitors with collective specificity for a variety of PTPs, including SHP-1, SHP-2, LYP, TCPTP, PTP1B and HePTP, failed to cause a measurable increase in CD3-induced p-ERK induction (Supplementary Fig. 4c). Likewise, for two PTPs, namely TCPTP and SHP-1, lower p-ERK induction in memory CD8^+^ T cells compared with naïve T cells in response to S-αCD3 applied to cells from *ptpn2*^*−/−*^ and *shp1*^*+/−*^ mice, respectively (Fig. 4g,h); note that *shp1*^*−/−*^ mice were not tested because of the T-cell developmental defect in these mice.

### An important role for CD45

**Figure 5:**
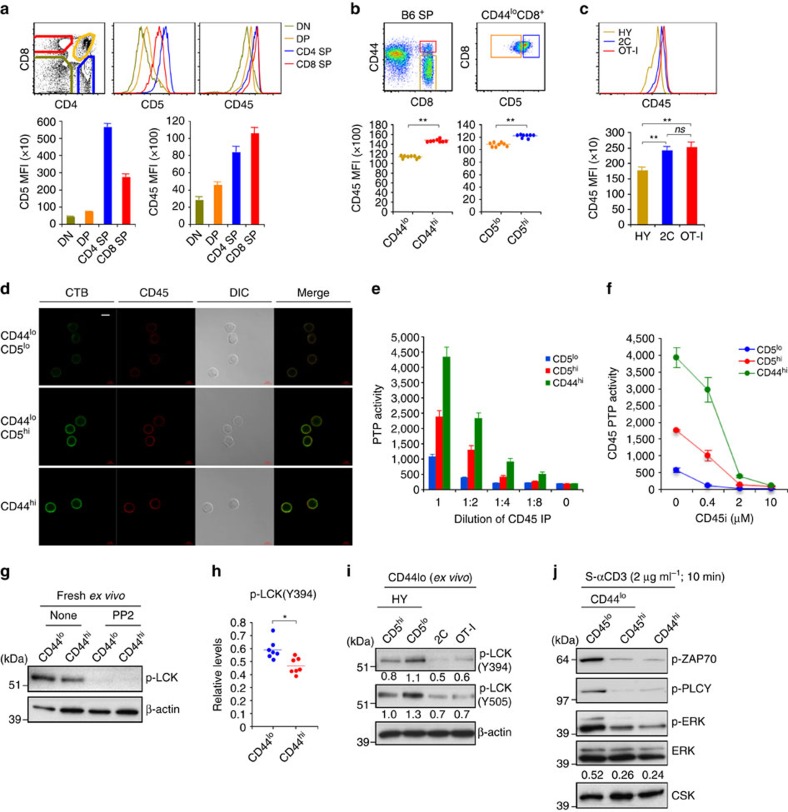
Expression of CD45 and its PTP activity in CD8^+^ T cell subsets. (**a**) Expression levels for CD5 and CD45 (top) and MFI levels (bottom) on the indicated subsets of B6 thymocytes (means±s.d.; *n*=6). (**b**) Levels of CD45 expression on CD44^lo^ and CD44^hi^ (bottom left) or CD44^lo^ naïve CD5^lo^ and CD5^hi^ cells (bottom right) gated for CD8^+^ T cells from B6 SP (top; *n*=8). (**c**) Levels of CD45 in HY, 2C and OT-I naïve CD8^+^ T cells (means±s.d.; *n*=5). (**d**) Confocal staining for CD45 and CTB (to detect lipid rafts) in CD44^lo^ naïve CD5^lo^ and CD5^hi^ cells and CD44^hi^ MP CD8^+^ T cells (original magnification × 63; × 1.4 zoom; scale bars, 5 μm). (**e**,**f**) *In vitro* PTP activity of CD45 immunoprecipitated by anti-CD45 mAb with whole cell lysates from equal numbers of CD44^lo^ naïve CD5^lo^ and CD5^hi^ cells and CD44^hi^ MP CD8^+^ T cells without (**e**) and with indicated concentrations of CD45-specific inhibitor, CD45i (**f**) (means±s.d.). (**g**,**h**) Expression (**g**, Western blot; **h**, densitometric levels relative to β-actin) of tyrosine-phosphorylated LCK (Y394) with or without LCK inhibitor PP2 (5 μM) in freshly isolated CD44^lo^ and CD44^hi^ B6 CD8^+^ T cells. (**i**) Levels of basal p-Y394-LCK and p-Y505-LCK in freshly isolated HY (CD5^lo^), 2C and OT-I naïve CD8^+^ T cells; note that CD5^hi^ HY cells are TCR-clonotype negative. (**j**) Levels of phosphorylated ZAP-70, PLCγ, and ERK in CD44^lo^ naïve CD45^lo^ and CD45^hi^ cells and CD44^hi^ MP CD8^+^ T cells after incubation with S-αCD3 mAb. Data are representative of two (**a**,**c**,**d**), at least three (**b**,**e**,**f**,**i**,**j**) and seven independent experiments (**g**,**h**). Unpaired Student's *t*-test was used for the statistical analysis. *P<0.005; **P<0.0005; *ns,* not significant.

**Figure 6:**
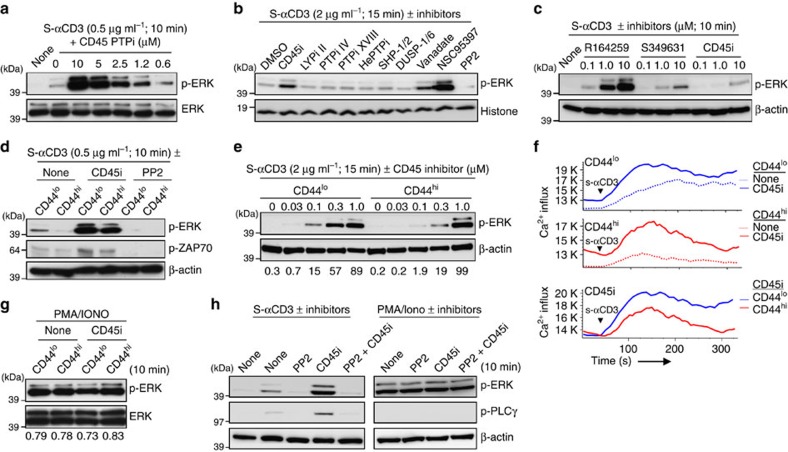
Effects of CD45 inhibitors on TCR sensitivity of CD8^+^ T cell subsets. (**a**) ERK phosphorylation in B6 naïve CD8^+^ T cells after incubation with S-αCD3 mAb±CD45 PTP inhibitor (CD45i). (**b**) ERK phosphorylation in B6 naïve CD8^+^ T cells after incubation with S-αCD3 mAb±various PTP inhibitors indicated (5–10 μM); note that increased p-ERK was observed only with CD45-specific inhibitors, CD45i and NSC95397, and as a control, vanadate. (**c**) ERK phosphorylation in B6 naïve CD8^+^ T cells after incubation with S-αCD3 mAb (0.5 μg ml^−1^)±CD45-specific inhibitors, R164259, S349631 and CD45i. (**d**,**e**) ERK (**d**,**e**) and ZAP-70 (**d**) phosphorylation in CD44^lo^ and CD44^hi^ B6 CD8^+^ T cells after incubation with S-αCD3 mAb±CD45i (**d**,**e**), or PP2 (**d**). (**f**) Ca^2+^ flux in CD44^lo^ (top) and CD44^hi^ (middle) CD8^+^ T cells gated from Indo-1-loaded B6 LN cells in response to S-αCD3 and S-αCD8 mAbs (10 μg ml^−1^)±CD45i (2 μM). Ca^2+^ flux for both subsets with CD45i treatment is shown as overlay (bottom). (**g**) ERK phosphorylation in CD44^lo^ and CD44^hi^ B6 CD8^+^ T cells after incubation with PMA/IONO±CD45i (10 μM). (**h**) ERK and PLCγ phosphorylation in B6 naïve CD8^+^ T cells after incubation with S-αCD3 mAb (2 μg ml^−1^) or with PMA/ionomycin±PP2 (2 μM), CD45i (5 μM), or both. Data are representative of four (**a**,**b**,**d**), three (**c**,**g**,**h**) and two independent experiments (**e**,**f**).

Despite the above negative findings on PTP inhibitors, we turned our attention to another PTP, namely CD45. CD45 is expressed ubiquitously on lymphoid cells and plays a key role in regulating TCR signalling by inducing tyrosine dephosphorylation of p56^lck^ (LCK) Y394 (activating motif) and also Y505 (inhibitory motif)^30^; in post-thymic T cells, phosphorylation of Y394 is most important because pY394/pY505 double-phosphorylated LCK has the same functional kinase activity as LCK pY394 alone^31^. For the following reasons, elevated expression of CD45 has emerged as a likely explanation for the decreased TCR sensitivity of CD44^hi^ memory CD8^+^ T cells, and also of CD44^lo^ naïve CD5^hi^ cells versus CD5^lo^ cells:

First, as for CD5, levels of CD45 were higher on mature CD4^+^ and CD8^+^ SP thymocytes than on CD4^+^CD8^+^ DP precursor cells^32^ (Fig. 5a), consistent with TCR tuning of thymocytes. Second, for naïve CD44^lo^ CD8^+^ T cells, including cells from TCR Tg strains, levels of CD5 correlated directly with CD45 levels, both for total CD45 levels (Fig. 5b bottom right, c) and expression of the different isoforms of CD45 (Supplementary Fig. 5a). Third, CD45 expression was clearly higher on memory cells than naïve cells, both for MP cells (Fig. 5b, bottom left) and induced memory cells (Supplementary Fig. 5b,c). This difference in CD45 levels was confirmed by confocal staining (Fig. 5d) and, notably, was in direct accord with CD45 phosphatase activity (Fig. 5e,f and Supplementary Fig. 5d); to measure the latter, total CD45 was immunoprecipitated by anti-CD45 Abs from CD8^+^ T-cell lysates and PTP activity was then assessed *in vitro* with a specific CD45 inhibitor^33^,^34^ (CD45i; N-(9,10-Dioxo-9,10-dihydro-phenanthren-2-yl)-2,2-dimethyl-propionamide) (Supplementary Fig. 5e,f). Fourth, significantly, high CD45 expression and its phosphatase activity correlated directly with reduced constitutive levels of its major substrate p-Y394-LCK, and also p-Y505-LCK, in memory cells (Fig. 5g,h and Supplementary Fig. 5g,h); this also applied for CD5^hi^ versus CD5^lo^ B6 naïve CD8^+^ T cells (Fig. 5i and Supplementary Fig. 5g,h; for Fig. 5i, data are shown also for HY naïve subsets, with unseparated naïve 2C and OT-I cells as a control). Fifth, for naïve CD8^+^ T cells, sorted cells expressing a high density of CD45 (CD45^hi^), like CD5^hi^ cells, exhibited weaker CD3-induced TCR signalling (p-ZAP-70, p-PLCγ and p-ERK) than CD45^lo^ cells (Fig. 5j and Supplementary Fig. 5i). Finally, and most importantly, the above-mentioned specific inhibitor of CD45 phosphatase activity, CD45i (Fig. 5f), caused a prominent increase in p-ERK induction following CD3 ligation (Fig. 6a); this applied to three additional CD45-specific inhibitors (NSC95397, R164259 and S349631)^35^ with a similar and even more marked enhancing effect relative to CD45i (Fig. 6b,c and Supplementary Fig. 6a,b); no such effect was seen with a spectrum of inhibitors reactive to other PTPs (Fig. 6b). The enhancing effect of CD45i applied to both naïve and memory CD8^+^ T cells (Fig. 6d) and depended on the relative level of CD45 on the cells; thus, titration experiments showed that the enhancing effect of CD45i was less pronounced on CD44^hi^ than CD44^lo^ cells (Fig. 6e) and, for naïve cells, less on CD44^lo^ CD5^hi^ cells than CD44^lo^ CD5^lo^ cells (Supplementary Fig. 6c) and also less on CD44^lo^ CD45^hi^ cells than CD44^lo^ CD45^lo^ cells (Supplementary Fig. 6d). In addition to p-ERK, CD45i enhanced p-ZAP-70 induction (Fig. 6d) and also led to an augmented Ca^2+^ flux (Fig. 6f).

Unlike CD3 ligation, the enhancing effect of CD45i was not observed with stimulation via PMA and ionomycin or IL-2 (Fig. 6g and Supplementary Fig. 6e) and was abolished by adding an inhibitor (PP2) of LCK (Fig. 6h and Supplementary Fig. 6f), implying that CD45i selectively enhanced TCR signalling events. When tested on B cells as a specificity control, CD45i reduced (rather than enhanced) p-ERK induction in response to IgM ligation, with little or no effect of adding PP2 (Supplementary Fig. 6g). Collectively, these findings indicate that the enhancing effects of CD45i on TCR signalling of CD8^+^ T cells are largely specific and LCK dependent.

### Influence of CD45 inhibition in basal TCR signalling

**Figure 7:**
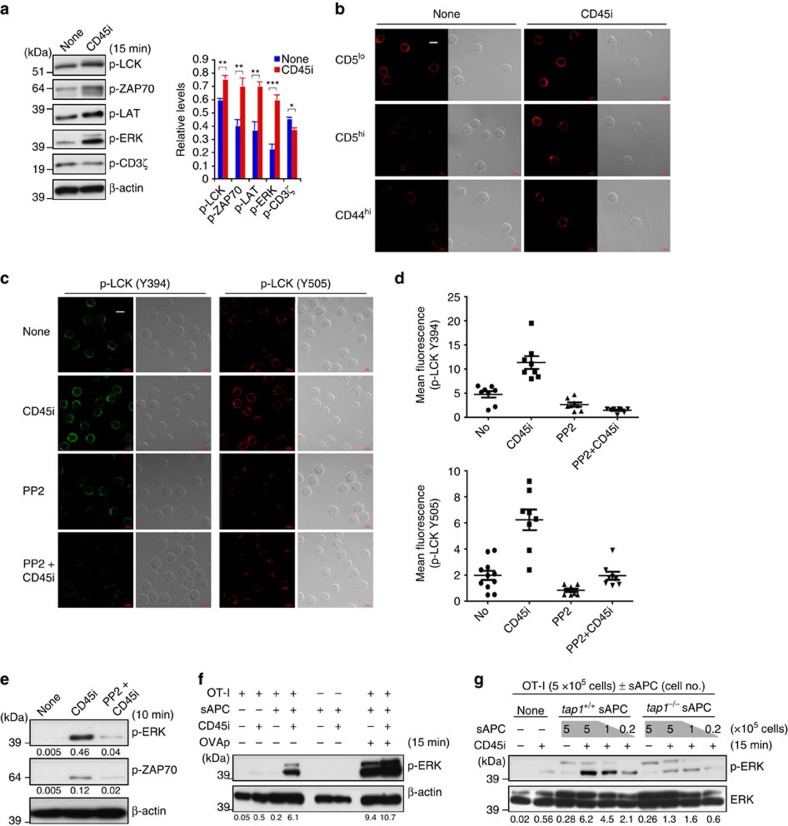
Influence of CD45 blockade on basal TCR signalling. (**a**) Levels of phosphorylated LCK, ZAP-70, LAT, ERK, and CD3ζ in purified unstimulated CD44^lo^ B6 CD8^+^ T cells with or without brief culture with CD45i (5 μM; means±s.d.). (**b**) Confocal staining for p-Y394-LCK in freshly isolated CD44^lo^ naïve CD5^lo^ and CD5^hi^ cells and CD44^hi^ MP CD8^+^ T cells at 15 min after incubation with or without CD45i (5 μM; original magnification × 63; × 1.4 zoom; scale bars, 5 μm). (**c**) Confocal staining for p-Y394-LCK and p-Y505-LCK in freshly isolated naïve CD8^+^ T cells at 15 min after incubation with or without PP2 (2 μM), CD45i (5 μM) or both (original magnification × 63; × 1.4 zoom; scale bars, 5 μm). (**d**) MFI levels for p-LCK per each cell visualised by confocal staining as in (**c**) (means±s.d.). (**e**) ERK and ZAP-70 phosphorylation in unstimulated B6 naïve CD8^+^ T cells with or without CD45i (5 μM)±PP2 (2 μM). (**f**) Naïve OT-I CD8^+^ T cells either untreated or briefly treated with CD45i (5 μM) were incubated with or without sAPC±OVAp (0.1 μM) and analysed for ERK phosphorylation. (**g**) Naïve OT-I CD8^+^ T cells either untreated or treated with CD45i (5 μM) were incubated with or without indicated numbers of *tap1*^*+/+*^ and *tap1*^*−/−*^ sAPC and analysed for ERK phosphorylation. Data are representative of three (**a**,**e**–**g**) and two independent experiments (**b**–**d**). Unpaired Student's *t*-test was used for the statistical analysis. *P<0.05; **P<0.005; ***P<0.0005.

The above finding that CD45 inhibition enhanced CD3-induced TCR signalling raises the question whether steady-state levels of CD45 on resting CD8^+^ T cells control quiescent basal TCR signalling. Here, it is striking that, even without CD3 ligation, adding CD45i alone to purified B6 naïve CD8^+^ T cells caused a rapid increase in background expression of p-ERK and also p-LCK, p-ZAP-70 and p-LAT, though not p-CD3ζ, implying onset of TCR signalling (Fig. 7a); likewise, CD45i caused an increase in background Ca^2+^ influx, especially in naïve CD8^+^ T cells (Supplementary Fig. 7a). The enhancing effect of CD45i on resting CD8^+^ T-cell subsets was confirmed by immunoblot analysis and elevated confocal staining for p-Y394-LCK for both naïve (CD5^lo^ and CD5^hi^) cells and CD44^hi^ MP cells (Fig. 7b and Supplementary Fig. 7b); similar findings applied for p-Y505-LCK (Fig. 7c,d, tested on naïve cells) and also p-ZAP70 (Supplementary Fig. 7c). Importantly, as for CD3 ligation, the enhancing effect by CD45i alone on basal TCR signalling (p-LCK, p-ZAP-70 and p-ERK) was abolished by adding PP2 (Fig. 7c–e and Supplementary Fig. 7c), indicating dependency on LCK.

The above enhancing effect of CD45i on basal TCR signalling was observed in the absence of APC (though it should be noted that CD8^+^ T cells themselves are MHC I^+^). Notably, however, with naïve OT-I CD8^+^ T cells, the increase in background p-ERK induced by CD45i was greatly increased by addition of APC, with or without specific peptide (Fig. 7f). This enhancing effect by adding APC was much less prominent with MHC-I^lo^ Tap1-deficient APC (Fig. 7g), implying a requirement for TCR contact with self-pMHC ligands on APC. The implication therefore is that basal TCR signalling driven by tonic TCR contact with self-pMHC on APC is continuously kept in check by CD45 PTP activity via modulation of active LCK, thereby maintaining self-tolerance; such tolerance is broken by addition of CD45i.

**Figure 8:**
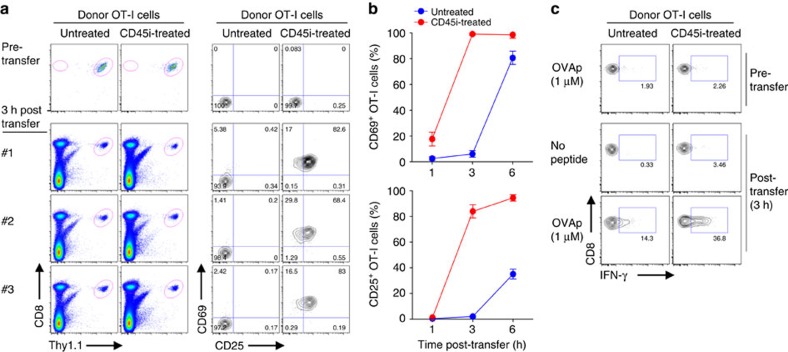
Effects of CD45 inhibition on TCR sensitivity to foreign antigen. (**a**) Naïve OT-I CD8^+^ T cells (Thy1.1) either untreated or pretreated with CD45i (5 μM) for 30 min *in vitro* were injected i.v. into B6 mice (*n*=3) that had been preinjected i.p. with OVAp (5 μg) plus polyI:C (10 μg) 2 h before adoptive transfer. At 3 h after transfer, spleens were collected and analysed by flow cytometry for expression of early activation markers, CD69 and CD25, on donor OT-I cells. (**b**) Mice as in **a** were analysed at 3 and 6 h after adoptive transfer for percentage of donor OT-I cells expressing CD25 or CD69 (means±s.d.). (**c**) Spleen cells collected at 3 h after transfer as in **a** were cultured *in vitro* with or without OVAp for 5 h and analysed by flow cytometry for intracellular IFN-γ production. Data are representative of two independent experiments (**a**–**c**).

The ability of CD45 to restrain basal TCR signalling raises the possibility that inhibiting CD45 activity on CD8^+^ T cells would enhance TCR sensitivity to foreign antigens. In support of this possibility, naïve OT-I cells treated briefly *in vitro* with CD45i and then adoptively transferred to B6 mice showed much faster upregulation of early activation markers, CD25 and CD69, following OVA peptide immunization, as compared with untreated OT-I cells (Fig. 8a,b); likewise, downregulation of CD62L and TCRβ was markedly enhanced in CD45i-treated OT-I cells (Supplementary Fig. 8a), Similar findings applied to IFN-γ production (Fig. 8c and Supplementary Fig. 8b,c). These data required the presence of specific peptide. Hence, although CD45i pretreatment did not induce overt self-reactivity, such treatment did substantially augment TCR sensitivity to a foreign antigen.

Collectively, these findings indicate that high expression of CD45 on peripheral CD8^+^ T-cell subsets in steady-state condition is an important checkpoint for modulating their relative TCR sensitivity, not only to self-ligands but also to foreign antigens.

## Discussion

The data in this paper shed new light on TCR tuning. As mentioned earlier, TCR tuning is well documented during thymic development and is viewed as a mechanism to fine-tune the TCR sensitivity of mature post-thymic T cells. For naïve T cells, these cells have to receive TCR signals through contact with self-pMHC ligands to survive in interphase, but such signalling has to be relatively weak to avoid breaking self-tolerance. Although TCR tuning is associated with upregulation of a number of negative regulators of TCR signalling, notably CD5, the precise mechanism of TCR tuning is still unclear. As shown here, addressing this question depends on the particular parameters used to measure TCR sensitivity.

In initial experiments, we found no evidence that mature T cells with high levels of CD5 displayed a reduction in TCR sensitivity. In fact, by three parameters, the TCR reactivity of CD5^hi^ naïve CD8^+^ T cells was actually higher than for CD5^lo^ cells. This applied to constitutive phosphorylation of CD3ζ, to *in vitro* proliferation induced by CD3 ligation, and, as shown previously^12^, to HP occurring in lymphopenic hosts. Moreover, as for HP, recent studies showed that CD5^hi^ naïve T cells led to far greater antigen-specific expansion than CD5^lo^ cells after pathogen infection^10^,^13^. For early TCR signalling events, however, the results described here were totally different. Thus, as defined by induction of p-ERK, p-ZAP-70, p-PLCγ, a Ca^2+^ flux and CD69 expression soon after CD3 ligation, CD5^hi^ cells were appreciably less TCR sensitive than CD5^lo^ cells. Hence, by these parameters, CD5^hi^ cells did display clear evidence of reduced TCR sensitivity relative to CD5^lo^ cells. The opposite results observed for later (∼2–3 d) proliferative responses can be attributed to differences in sensitivity to cytokines. Thus, correlating with enhanced expression of GM1-containing lipid rafts, CD5^hi^ cells were more sensitive than CD5^lo^ cells to stimulation by endogenous IL-2 produced following CD3 ligation *in vitro*, and also to the raised levels of IL-7 that drive HP in lymphopenic hosts^12^. The increased background expression of p-CD3ζ by CD5^hi^ cells is less easy to explain. The possibility we favour is that the partial phosphorylation of CD3ζ induced by continuous TCR interaction with self-pMHC ligands leads to a weak form of TCR signalling which selectively promotes sensitivity to cytokines, thereby making CD5^hi^ cells more sensitive to IL-7 and IL-2 than CD5^lo^ cells. But we envisage that such signalling is not sufficient to induce typical signs of TCR signalling such as p-ERK induction. On this point, high constitutive p-CD3ζ expression on CD5^hi^ cells is not accompanied by measurable background expression of p-ERK, implying a lack of downstream TCR signalling.

As for naïve T cells, defining the TCR sensitivity of memory CD8^+^ T cells depended on the assay used. As mentioned earlier, most, though not all, previous studies found that memory CD8^+^ T cells gave more prominent proliferative responses to antigen than naïve cells. In line with this finding, as shown here, MP CD44^hi^ CD8^+^ T cells gave far stronger *in vitro* proliferative responses after CD3 ligation than CD44^lo^ cells, reflecting both higher synthesis of IL-2 and higher expression of IL-2Rβ (CD122) by CD44^hi^ cells than CD44^lo^ cells. Likewise, the observation that HP in lymphopenic hosts is faster and more intense for CD44^hi^ cells than CD44^lo^ cells^36^ may reflect the enhanced sensitivity of CD44^hi^ cells to IL-7; as for IL-2, this enhanced sensitivity may in part reflect the more open configuration of cytokine loci in memory T cells^37^. Despite these findings, early TCR signalling events were clearly less marked in CD44^hi^ cells than CD44^lo^ cells. This finding applied to induction of p-ERK, p-ZAP-70, p-PLCγ, a Ca^2+^ flux and upregulation of CD69, and was apparent for both MP and induced memory CD8^+^ T cells and with stimulation by peptide-loaded APC as well as by CD3 or CD3/CD28 ligation. By these parameters, memory CD8^+^ T cells showed reduced TCR sensitivity relative to naïve CD8^+^ T cells. It is important to emphasize that this evidence for TCR desensitisation of memory cells applied only within the first few hours of stimulation and was reversed at later stages as the result of stimulation by endogenous IL-2.

With regard to the cause of TCR desensitisation in memory CD8^+^ T cells, regulation by CD5 seems unlikely because, in contrast to MP cells, TCR transgenic Ag_MEM_ and HP_MEM_ CD44^hi^ cells both showed substantially lower expression of CD5 than naïve cells. Likewise, based on studies with gene knockout mice, we could find no evidence that the decreased TCR sensitivity of memory CD8^+^ T cells reflected increased expression of CBL-B. Others have described lower TCR expression on memory cells than naïve cells^19^. However, in our hands this difference, though detectable, was very slight (Supplementary Fig. 5b).

For PTPs, others reported increased expression of PTPN2 (TCPTP), PTPN12 (PTP-PEST) and PTPN22 (LYP) in memory CD8^+^ T cells^19^, which is of interest because deletion of these and other PTPs is associated with TCR hyper-reactivity and onset of autoimmune disease^28^,^29^,^38^,^39^,^40^,^41^. In our studies, we found increased expression of SHP-1 and SHP-2 proteins, as well as TCPTP and LYP proteins both in MP cells and CD5^hi^ naïve CD8^+^ T cells relative to CD5^lo^ cells. However, it is questionable whether the elevation of these and other PTPs could account for the decreased TCR sensitivity in these cells. If this were the case, broad-spectrum phosphatase inhibitors would be expected to increase TCR sensitivity. However, tests with six different phosphatase inhibitors showed no increase in TCR signalling when the inhibitors were added during CD3 ligation. Consistent with the inhibitor data, we could find no evidence for a role of TCPTP or SHP-1 in TCR tuning of CD8^+^ T cells when cells lacking TCPTP or expressing reduced levels of SHP-1 were tested. The implication therefore is that, though important for modulating the immune response, the above individual PTP may have only a minor role in restraining TCR signalling of resting CD8^+^ T cells. Vanadate (Na_3_VO_4_), a potent inhibitor for multiple PTPs, did show a clear enhancing effect of p-ERK induction after CD3 ligation. However, this effect may be largely a reflection of inhibition of CD45 activity (see below).

The PTP inhibitors that failed to alter TCR signalling sensitivity included reagents with specificity for PTPN22. This finding may seem surprising because deficiency of PTPN22 was recently shown to cause enhanced TCR sensitivity to stimulation with relatively weak antigenic peptides, though this effect was not seen with strong peptides^28^. However, in our studies the differences in TCR sensitivity between naïve and memory cells and also between CD5^hi^ and CD5^lo^ naïve cells applied equally to strong and weak TCR stimulation. Moreover, for naïve cells, we observed only a marginal difference in PTPN22 levels between CD5^hi^ and CD5^lo^ cells despite their prominent difference in TCR sensitivity. It should be mentioned that in other studies PTPN22 deficiency had little or no effect on TCR sensitivity under various conditions^38^,^42^. Despite these findings, PTPN22-deficient mice do show a moderate increase in memory CD8^+^ T cells in later life, though with no change in naïve cells^38^. Hence it seems likely that PTPN22 does play a significant, though mild role in restricting TCR sensitivity to self-ligands.

With regard to other PTPs, PTPN2 clearly deserves consideration because PTPN2-deficient CD8^+^ T cells were reported to be more reactive than wild-type (WT) cells in response to TCR ligation *in vitro*, and gave far stronger HP in lymphopenic hosts^27^,^41^. Consistent with these data, we found that PTPN2-deficient cells showed relatively higher p-ERK induction than WT cells after CD3 ligation. Nevertheless, the hierarchy of TCR sensitivity reported here for CD8^+^ T cells (CD44^lo^ CD5^lo^>CD44^lo^ CD5^hi^>CD44^hi^) was the same for PTPN2-deficient and WT cells. Similar findings applied for *shp1*^*+/−*^ versus *shp1*^*+/+*^ cells. Hence, altered expression of these two PTPs seems an unlikely explanation for the difference in TCR sensitivity shown here for resting CD8^+^ T-cell subsets.

In searching for other explanations for TCR tuning of peripheral CD8^+^ T cells, the possibility that CD45 could be involved may seem unlikely because CD45 is generally viewed as a positive regulator of TCR signalling by causing dephosphorylation of inhibitory p-Y505-LCK (ref. 43). However, more recent evidence suggests that, for mature T cells, CD45 has an overall negative effect on TCR signalling by dephosphorylating p-Y394-LCK, which keeps LCK in an active configuration. In favour of a negative effect of CD45, several studies with transgenic mice have shown that reducing total amounts or relative activity of CD45 on T cells to 10–40% of normal can cause TCR hyper-reactivity, accelerated rejection of pathogens, and signs of autoimmunity^33^,^44^,^45^,^46^. Likewise, there is reciprocal evidence that overexpression of CD45 in transgenic mice reduces TCR reactivity^47^,^48^. The data shown here on CD8^+^ T-cell subsets from B6 mice are clearly consistent with this model. Thus, for naïve CD8^+^ T cells, the reduced TCR sensitivity of CD5^hi^ cells relative to CD5^lo^ cells correlated directly with higher expression of total CD45 (and the various CD45 isoforms), higher levels of total CD45 phosphatase activity, and reduced expression of p-Y394-LCK (and also p-Y505-LCK). Similarly, the decreased TCR sensitivity of memory CD8^+^ T cells versus naïve CD8^+^ T cells correlated with higher CD45 levels (and also total CD45 PTP activity) and lower p-Y394-LCK on memory cells. One apparent discrepancy is that, in sharp contrast to CD45 transgenic mice with intermediate levels of CD45, T cells from CD45 transgenic mice with unphysiologically low levels of CD45 (∼3–15% of WT levels) showed reduced TCR sensitivity^45^,^48^. This finding correlated with aberrantly high accumulation of inactive p-Y505-LCK on the cells and may have no counterpart in normal mice where CD45 levels are substantially higher. Thus, as shown here in normal mice, mature T cells with the lowest density of CD45 invariably had the highest TCR sensitivity.

Studies with CD45i provided direct support for the view that the reduced TCR sensitivity of CD5^hi^ naïve and memory CD8^+^ T cells, relative to naïve CD44^lo^ CD5^lo^ T cells, reflected negative regulation by CD45. Thus, in marked contrast to the other PTP inhibitors tested, addition of CD45i greatly increased p-ERK and p-ZAP-70 induction and also a Ca^2+^ flux after CD3 ligation, both for naïve and memory CD8^+^ T cells. Although PTP inhibitors are rarely if ever entirely specific for particular substrates, multiple approaches suggested that the specificity of CD45i was heavily skewed to CD45 with minimal activity on other candidate modulators of TCR signalling. In particular, the capacity of CD45i to enhance TCR signalling was observed with three other inhibitors with known CD45-specific reactivity but was undetectable with other PTP inhibitors. Moreover, CD45i was highly specific for TCR/CD3-induced, LCK-dependent signalling. Thus, the enhancing ability of CD45i was (a) abrogated by addition of an LCK inhibitor PP2, (b) did not apply to stimulation of T cells via PMA and ionomycin or IL-2 and (c) also did not apply to IgM-induced stimulation of B cells (where CD45 acts as a positive regulator for BCR signalling^48^). In addition, it is notable that the enhancing effect of CD45i on CD8^+^ T cells was dependent on the density of CD45 on the cells, being more marked for cells with a low density of CD45, that is, CD44^lo^ naïve cells sorted for low CD45 expression. Collectively, these data validate the credentials of CD45i for inhibiting CD45 activity and thereby consolidate the view that the restraining influence of CD45 on TCR sensitivity is most prominent for CD8^+^ T cells with the highest density of CD45, namely memory cells.

Although the capacity of high CD45 levels to limit TCR sensitivity was shown previously for CD45 transgenic mice^45^,^48^, the data presented here provide the first clear evidence that levels of CD45 on T cells are physiologically relevant and control the relative TCR sensitivity of resting CD8^+^ T cells, both for naïve and memory subsets. For both subsets, CD45 levels correlated directly with levels of p-Y394-LCK, thereby serving to modulate proximal TCR signalling events and reduce self-reactivity. For naïve CD8^+^ T cells, CD45 levels correlated directly with CD5 levels. Since CD5 expression on CD8^+^ T cells is thought to correlate directly with their intrinsic TCR/self-pMHC reactivity, the implication therefore is that cells with relatively strong self-reactivity (CD5^hi^ CD3ζ^hi^ CD45^hi^ CD8^+^ T cells) are restrained from overt self-responsiveness by their high density of CD45, which reduces p-Y394-LCK and thereby limits TCR sensitivity. In this respect, it is notable that addition of CD45i to purified CD8^+^ T cells without CD3 ligation led to p-ERK induction and other signs of TCR signalling, presumably through TCR contact with self-pMHC ligands on neighbouring T cells. Moreover, much stronger p-ERK induction occurred with addition of APC, but not with MHC-I^lo^ Tap1-deficient APC. Consistent with the effect of CD45i on increasing TCR sensitivity, pretreating OT-I cells with CD45i before adoptive transfer markedly enhanced the early response of these cells to specific peptide. Hence, CD45i treatment increases responsiveness to both self and foreign antigens. In future studies it will be important to extend these findings by examining the effects of conditionally deleting CD45 in mature T cells.

Although CD45 levels correlated closely with p-Y394-LCK levels, precisely how CD45 controls TCR tuning is unclear. Various factors could be important, including distribution/localization of CD45 to lipid rafts on the T-cell membrane, protein interaction via the CD45 cytoplasmic domain, different ligand interaction via differential glycosylation of its extracellular domain, and variation in dimerization by different isoforms^49^. It is also unclear how the density of CD45 on mature CD8^+^ T cells is controlled. For resting memory CD8^+^ T cells, it is notable that survival of these cells, unlike naïve cells, is MHC-I independent, implying an absence of baseline TCR signalling in memory cells. However, despite the apparent inverse correlation between CD45 expression and tonic TCR signalling, understanding this relationship will require further investigation.

In conclusion, we show here that, after positive selection in the thymus, naïve CD8^+^ T cells are subjected to a second step of TCR tuning via upregulation of CD45, which reduces basal levels of p-Y394-LCK and thereby modulates their TCR sensitivity. CD45 upregulation is most prominent for cells with high intrinsic self-pMHC reactivity and is tailored to enable naïve cells to receive survival signals through TCR/self-pMHC interaction while maintaining self-tolerance, but without compromising reactivity to foreign antigens. A further step of TCR tuning occurs when naïve CD8^+^ T cells respond to antigen and become memory cells: enhanced CD45 upregulation on memory cells mildly reduces their TCR sensitivity (and self-reactivity) but elevated responsiveness to cytokines and possibly also other mediators ensure that these cells display robust responses to foreign antigens. In addition to CD45, TCR sensitivity is probably modulated by other PTPs and/or other negative regulators on T cells; whether the latter inhibitors act during normal T-cell homeostasis rather than during the immune response is still unclear.

## Methods

### Mice

C57BL/6 (B6) and B6.SJL (Ly5.1) mice were obtained from Australian Bioresources Centre (ABR, Australia) and POSTECH Biotech Centre (PBC, Korea). Sources of OT-I (Thy1.1), 2C (Ly5.1) and HY TCR Tg mice, and *il2*^*−/−*^ and *tap1*^*−/−*^ mice, all on a B6 background, were reported^12^. The *cblb*^*−/−*^ homozygote and *cblb*^*+/+*^ WT mice were all on a B10.BR background^50^ and obtained from the same littermates by crossing *cblb*^*+/−*^ heterozygote mice. *Lck-Cre.Ptpn2*^*fl/fl*^ (*ptpn2*^*−/−*^; conditional deletion for PTPN2) and motheaten viable mice (me^v^; null mutation for SHP-1), all on a B6 background, were used as reported^41^,^51^. All mice were maintained under specific pathogen-free conditions and female mice were used at 6–12 weeks of age for experiments without need of randomization or blinding tests, according to protocols approved by the Animal Experimental and Ethic Committee at the Garvan Institute (Australia) and the Institute for Basic Science (Korea).

### Reagents

Recombinant mouse IL-2 and IL-7 were purchased from PeproTech. SIYRp (SIYRYYGL; specific for 2C TCR) and OVAp (SIINFEKL) and its variant peptides (Q4R7, SIIQFERL; T4, SIITFEKL; and Q4H7, SIIQFEHL; all specific for OT-I TCR) were purchased from Mimotopes. LCK inhibitor PP2 and various PTP inhibitors, LYPi II, PTPi IV, PTPi XVIII, HePTPi, SHP1/2i, CD45i, NSC95397 and vanadate (Na_3_VO_4_) were all purchased from Calbiochem and dissolved in DMSO (Sigma-Aldrich). Two CD45-specific inhibitors, R164259 and S349631^35^, were generously provided by N. Bottini (La Jolla Institute for Allergy and Immunology).

### Antibodies for flow cytometry

Cell suspensions were prepared and stained for FACS analysis of cell-surface markers using PBS containing 2% FBS and 0.05% sodium azide with the following fluorochrome-conjugated Abs to (from BD Biosciences and eBioscience): CD4 (GK1.5 and RM4–5), CD5 (53-7.3), CD8α (53-6.7), CD27 (LG.7F9), CD43 (1B11), CD44 (IM7), CD45RA (14.8), CD45RB (16A), CD45RC (DNL-1.9), pan-CD45 (30-F11), CD45.1 (A20), CD62L (MEL-14), CD69 (H1.2F3), CD90.1 (HIS51), CD122 (TM-β1), CD127 (A7R34), CD183 (CXCR3-173) and Ly6C (HK1.4). All Abs were used at a concentration of 1:300–1:500. Flow cytometry samples were run using a LSR II or FACSCanto II (BD Biosciences) and analysed by FlowJo software (Tree Star).

### T- and B-cell purification

Pooled lymph node (LN) cells from the indicated mice were stained with fluorochrome-conjugated Abs to CD8α, CD5 and CD44, and then sorted to obtain CD44^lo^ CD5^lo^ and CD44^lo^ CD5^hi^ naïve CD8^+^ T cells and/or CD44^hi^ MP CD8^+^ T cells. The LN cells treated with Abs were also sorted to obtain total CD44^lo^ naïve and CD44^hi^ MP CD8^+^ T cells using a FACSAria (BD Biosciences) and Moflo-XDP (Beckman Coulter). For B cell purification, spleen cells stained with Abs to B220 (RA3-6B2; eBioscience; 1:300) and CD19 (1D3; eBioscience; 1:300) were sorted to obtain CD19^+^ B220^+^ mature splenic B cells. Purity was routinely tested after cell sorting and was>99%.

### Generation of Ag- or HP-induced memory CD8^+^ T cells

Naïve 2C CD8^+^ T cells (Ly5.1^+^) were isolated by a negative selection using MACS with a CD8^+^ T-cell isolation kit (Miltenyi Biotec) according to the manufacturer's instructions and transferred i.v. into irradiated (450 cGy) B6 mice (1 × 10^6^ cells per mouse). Recipient mice were either injected i.p. with SIYR peptide (10 μg) plus polyI:C (20 μg; Sigma-Aldrich) to generate Ag-induced memory cells (Ag_MEM_; *n*=5) or left unimmunized to generate HP-induced memory cells (HP_MEM_; *n*=4). At 56 days after adoptive transfer, SP and LN cells pooled from the recipient mice were first enriched for total CD8^+^ T cells by a MACS negative selection and then further isolated to obtain memory donor 2C CD8^+^ T cells by FACS sorting with >99% purity. As a control, naïve CD44^lo^ 2C CD8^+^ T cells were freshly isolated from LN cells of 2C TCR Tg mice by FACS. For generating OT-I memory cells, MACS-purified naïve OT-I CD8^+^ T cells (Thy1.1^+^) were transferred i.v. into either untreated (1 × 10^6^ cells per mouse; *n*=5) or irradiated B6 mice (*n*=8). On day 1 after adoptive transfer, untreated recipient mice were immunized i.v. with OVAp (10 μM)-pulsed B6 bone marrow-derived dendritic cells (BM-DC; 2 × 10^6^ cells for inducing Ag_MEM_); for inducing HP_MEM_, irradiated recipients of naïve OT-I cells were either left untreated (*n*=4) unless otherwise described or injected i.p. with IL-2 (1 μg)/αIL-2 mAb (S4B6; 10 μg; *n*=4) complexes^12^ to enhance proliferation. As with 2C memory cells, OT-I donor memory cells (both Ag_MEM_ and HP_MEM_) were purified at >6 months after adoptive transfer by FACS sorting. Naïve control CD44^lo^ OT-I cells were freshly isolated either directly from OT-I TCR Tg mice (*n*=3) or from B6 recipients adoptively transferred with naïve OT-I CD8^+^ T cells 7 days before (1 × 10^6^ cells per mouse; *n*=3).

### Measuring TCR sensitivity by CD3 ligation

For polyclonal CD8^+^ T cells, subsets of the indicated FACS-sorted naïve (total CD44^lo^, CD5^lo^ or CD5^hi^ cells) or memory (CD44^hi^) CD8^+^ T cells (0.5–2 × 10^6^ cells) were cultured for indicated various time points (ranging from 2 min to several hours for early and late responses, respectively) either with soluble (S) or plate-bound cross-linked (Cx) anti (α)-CD3 mAb (145-2C11; BD Biosciences; 0.5–5 μg ml^−1^ unless otherwise described)±S- or Cx-αCD28 mAb (37.51; BD Biosciences; 5 μg ml^−1^)±IL-2 (10 ng ml^−1^). For Ag-specific CD8^+^ T cells, OT-I TCR Tg CD8^+^ T cells (1–2 × 10^5^ cells) were incubated with T-cell-depleted irradiated (2,000 cGy) B6 splenic APC (sAPC; 0.5–1 × 10^6^ cells) pre-pulsed with 0.1 μM OVAp.

### TCR and BCR sensitivity with CD45 PTP inhibitor

FACS-sorted naïve and memory CD8^+^ T cells were preincubated for 10–30 min with indicated concentrations of either PP2 (2–5 μM), CD45i (0.03–10 μM) or both inhibitors and then cultured for additional 10–15 min with various stimuli, including S-αCD3 mAb (0.5–2 μg ml^−1^), PMA (50 ng ml^−1^) plus ionomycin (500 ng ml^−1^; Sigma-Aldrich) or high dose IL-2 (1 μg ml^−1^). In an experiment for assessing BCR sensitivity, FACS-purified splenic B cells were preincubated for 30 min with or without indicated inhibitors, PP2, CD45i or both, and then incubated for 10 min with goat anti-mouse IgM F(ab′)_2_ (10 μg ml^−1^; Jackson ImmunoResearch Laboratories). With addition of APC, sorted B6 or OT-I naïve CD8^+^ T cells pretreated with CD45i (5 μM) or a control PBS containing 0.1% DMSO were either left alone or coincubated with T-cell-depleted irradiated WC or Tap1-deficient sAPC (without CD3 mAb ligation); for some OT-I cultures with sAPC, OVAp (0.1 μM) was added as a positive control. For measuring TCR sensitivity to foreign antigens *in vivo*, OT-I naïve CD8^+^ T cells (Thy1.1) were pretreated with CD45i or PBS containing 0.1% DMSO for 30 min, washed twice, and transferred i.v. into B6 mice (1 × 10^6^ cells per mouse; *n*=3) that had been preinjected i.p. with OVAp (5 μg) plus polyI:C (10 μg) 2 h before adoptive transfer. At 3 and 6 h post-transfer, SP cells from the mice were analysed for expression of early activation markers, CD25 and CD69, and other various markers indicated, as well as for intracellular IFN-γ production after 5 h OVAp restimulation *in vitro*, by flow cytometry.

### Other phosphatase inhibitors

In experiments investigating the effects of other PTP inhibitors, FACS-sorted naïve B6 CD8^+^ T cells were cultured for 10 min with S-αCD3 mAb (0.5 μg ml^−1^) immediately after 30 min preincubation with titrated concentrations of the indicated PTP inhibitors, the specificity of the inhibitors (<10 μM IC_50_) according to the manufacturers being: LYPi II (TCPTP, PTP1B and PEP/LYP), PTPi IV (SHP-2, PTP1B and PTP-ɛ), PTPi XVIII (PTP1B, SHP-1 and YOP), HePTPi (TCPTP, PTP-SL, SHP-2, PTP1B, LYP, VHR, SHP-1, HePTP, MKP-3 and STEP), DUSP1/6i (DUSP1 and DUSP6) and SHP-1/2i (SHP-1, SHP-2, PTP1B and HePTP).

### Western blot

Purified naïve or memory CD8^+^ T cells cultured under the conditions indicated were collected, washed with ice-cold PBS and lysed on ice for 15–30 min in a lysis buffer (20 mM Tris, pH7.5, 150 mM NaCl, 1 mM EDTA, 1 mM EGTA, 1% Triton X-100, 2.5 mM sodium pyrophosphate, 1 mM β-glycerophosphate, 1 mM Na_3_VO_4_, 1 mM PMSF, 1 μg ml^−1^ aprotinin and leupeptin). Cell lysates were resolved by 4–12% bis–tris SDS–polyacrylamide gel electrophoresis Gel (Invitrogen), transferred onto nitrocellulose membrane (Invitrogen), blocked with 5% dry non-fat milk in Tris buffered saline (pH 7.4) containing 0.1% Tween-20, and probed with the following Abs to (mAbs were used at 1:1000 and purchased from Santa Cruz Biotechnology and Cell Signaling Technology unless otherwise described): phospho (p)-LCK (Tyr394; rabbit polyclonal), LCK (3A5), p-ZAP70 (Tyr319; 65E4), ZAP70 (IE7.2), p-LAT (Tyr191; E225; Abcam), LAT (FL-233; rabbit polyclonal), SLP76 (H-300; rabbit polyclonal), p-PLCγ (Tyr783; rabbit polyclonal), PLCγ1 (530; rabbit polyclonal), PKCθ (C-18; rabbit polyclonal; 1:500), p-ERK1/2 (Thr202/Tyr204; D13.14.4E), ERK1/2 (H-72; rabbit polyclonal), p-AKT (Ser473; 193H12), p-p38 (Thr180/Tyr182; D3F9), p-JNK (Thr183/Tyr185; 81E11), p-STAT5A/B (Tyr694/699; A11W; Millipore; 1:2,000), STAT5 (3H7), SHP-1 (C14H6), SHP-2 (D50F2), TCPTP (PTPN2; E-11 and M-115), LYP (PTPN22; G-3), Histone H3 (9715S; rabbit polyclonal) and β-actin (AC-15, AC-74; Sigma-Aldrich; 1:10,000). For detecting p-CD3ζ (p21 form), a relevant membrane section spanning a region between 14 and 38 kDa based on a protein size marker was cut and probed with Ab to p-Tyrosine (4G10; Millipore) and confirmed by stripping and reprobing with mAb to CD3ζ (1ζ3A1; BD Biosciences; 1:500). Immunoreactivity was detected by ECL detection system according to the manufacturer's instructions (GE Healthcare). Densitometry on the scanned blots was performed by histogram analysis using Photoshop CS3 Imaging Software (Adobe) for quantification of the band intensity and normalized to the control. Uncropped images of immunoblots are shown in Supplementary Fig. 9.

### CD45 immunoprecipitation and *in vitro* CD45 PTP assay

Equal numbers of FACS-purified B6 CD8^+^ T-cell subsets (CD44^lo^ naïve CD5^lo^ and CD5^hi^ cells and CD44^hi^ MP cells) were washed with ice-cold PBS and lysed on ice for 15–30 min in Pierce IP Lysis Buffer (Thermo Fisher Scientific) containing protease inhibitors. Cell lysates were incubated overnight with mAbs to CD45 (I3/2.3, 3H1379 or 30-F11; all from Santa Cruz Biotechnology; 1:500–1:1,000) and, as a control, mAb to CD5 (53-7.3; eBioscience; 1:250) and isotype mAb (rat IgG2b; eBioscience; 1:500), followed by Protein A/G PLUS-Agarose (Santa Cruz Biotechnology). For detecting phosphatase activity *in vitro*, the immunoprecipitated complexes were washed twice with IP lysis buffer and then twice with a PTP assay buffer (25 mM HEPES, 50 mM NaCl, 0.05% Tween 20, 1 mM dithiothreitol, pH 7.0) and were added with 50–100 μl PTP assay buffer. An indicated series of dilution of the immunoprecipitates was prepared and then incubated with 20 μM 6,8-difluoro-4-methylumbelliferyl phosphate (DiFMUP; Invitrogen) with or without various PTP inhibitors (5–20 μM) at 30 °C in a 96-well microtiter plate. Fluorescence (emission 450 nm and excitation 360 nm by the hydrolysed DiFMU) was measured from 30 to 60 min after reaction in a fluorescence-based microplate reader (Tecan Infinite F200 Pro). Background fluorescence of PTP assay buffer only was subtracted for each well.

### Confocal staining

For CD45 confocal staining, FACS-purified CD44^lo^ naïve CD5^lo^ and CD5^hi^ cells and CD44^hi^ MP CD8^+^ T cells were placed at 0.5–1 × 10^5^ cells on a poly-L-lysine-coated glass slide (Sigma-Aldrich), and allowed to adhere to the slide for 5 min at room temperature (RT). The cells were co-stained for 20 min on ice with fluorescein isothiocyanate-conjugated CTB (Sigma-Aldrich; 1:200) and Alexa Fluor 647-conjugated anti-CD45 (30-F11; Biolegend; 1:200) in PBS, washed twice with PBS and then fixed for 20 min with cold 4% paraformaldehyde in PBS without permeabilization. For confocal staining of LCK and ZAP-70, purified naïve CD8^+^ T cells were washed, fixed for 20 min with cold 4% paraformaldehyde in PBS, permeabilized for 5 min with 0.1% Triton X-100 in PBS and then blocked for 15 min with 5% normal goat serum in PBS containing 1% BSA. Cells were stained for 45 min with either unconjugated (for p-LCK) or Alexa Fluor 488-conjugated (for p-ZAP-70) primary Abs to p-Y394-LCK (Santa Cruz Biotechnology; 1:200), p-Y505-LCK (Santa Cruz Biotechnology; 1:200), and p-Y319-ZAP-70 (BD Bioscience; 1:200), washed, blocked, and then re-incubated for 30 min with Alexa Fluor 488-conjugated anti-rabbit (for p-Y394-LCK) or Alexa Fluor 647-conjugated anti-mouse (for p-Y505-LCK) IgG (Invitrogen; 1:200). The final slides were washed with PBS and mounted in ProLong Gold Antifade Reagent (Invitrogen) and analysed using a Zeiss LSM 700 laser scanning confocal microscope (Carl Zeiss) for acquiring fluorescence images.

### Ca^2+^ mobilization

Unsorted LN cells or FACS-purified CD8^+^ T-cell subsets from B6 mice (*n*=3) were labelled with 5 μM Indo-1 (Invitrogen) for 45 min at 37 °C followed by two washes and continued incubation at RT for staining with fluorochrome-conjugated Abs to CD8, CD5 and CD44 for additional 10 min. After a washing step, cell samples were prewarmed for 5 min at 37 °C and then run on a LSRII for 4–5 min before adding a stimulus to set baseline Ca^2+^ levels. Sample analysis was immediately continued by TCR stimulation with addition of S-αCD3 mAb (5 μg ml^−1^) for 3–4 min, either untreated or followed by adding cross-linking anti-hamster IgG (G94-56; BD Biosciences; 5 μg ml^−1^) for additional 2–3 min; for the latter cross-linking, incubation continued by adding ionomycin (1 μg ml^−1^) as a positive control for measuring saturated Ca^2+^ levels. Data are presented as the ratio of 398 nm (Indo-1 bound to Ca^2+^)/482 nm (unbound Ca^2+^) in the indicated subsets of CD8^+^ T cells.

### Intracellular staining for flow cytometry

For intracellular cytokine staining (ICS), cells stimulated with indicated stimuli in the presence of GolgiStop (BD Biosciences) were stained for cell-surface markers, fixed and permeabilized using Cytofix/Cytoperm buffer (BD Biosciences) and then stained with fluorochrome-conjugated Abs to IFN-γ (XMG1.2; eBioscience; 1:300) and TNF-α (MP6-XT22; BD Biosciences; 1:300) using Perm/Wash buffer (BD Biosciences). The same ICS protocol was used for analysing Bcl-2 and CD107a expression with fluorochrome-conjugated Abs to Bcl-2 (BCL/10C4; Biolegend; 1:200) and CD107a (1D4B; eBioscience; 1:300). Intracellular staining for T-bet and Eomes expression was performed with Foxp3 Staining Buffer Set (eBioscience) according to the manufacturer's instructions using fluorochrome-conjugated Abs to T-bet (4B10; 1:200) and Eomes (Dan11mag; all from eBioscience; 1:200). For intracellular staining for p-ERK, B6 SP cells treated with indicated stimuli were fixed with 2% paraformaldehyde at RT for 15 min, followed by permeabilization with ice-cold 90% methanol for 20 min on ice. After a washing step, cells were blocked with PBS containing 2% FBS and incubated with fluorochrome-conjugated Ab to p-ERK (Thr202/Tyr204; D13.14.4E; Cell Signaling Technology; 1:100), followed by repeated washes and continued incubation for 15 min on ice with fluorochrome-conjugated Abs to cell-surface markers.

### Cytokine ELISA

For detection of IL-2 and IFN-γ secretion, culture supernatants from cells incubated with indicated stimuli were collected and analysed by a standard protocol using a cytokine sandwich ELISA kit for IL-2 and IFN-γ (all from BD Biosciences) according to the manufacturer's instructions.

### CFSE labelling and proliferation

FACS-purified subsets of CD8^+^ T cells from mice indicated were labelled with CFSE (2 μM) as described previously^12^ and cultured for 2–7 days either with high doses of cytokines, IL-2 (0.1–1 μg ml^−1^) and IL-7 (1 μg ml^−1^), or with Cx-αCD3 mAb (0.1–10 μg ml^−1^); in some experiments with the latter TCR stimulation, a mixture of mAbs to IL-2 (JES6-1A12) and CD122 (TM-β1; 10 μg ml^−1^; all from BD Biosciences) were added to block IL-2 signalling. Cells were collected and CFSE dilutions were analysed by flow cytometry. Alternatively, proliferative responses of the indicated TCR-stimulated CD8^+^ T cells were analysed by adding [^3^H]thymidine (1 μCi per well) and after a 6–12 h pulse, cells were collected and measured by a β-counter (TopCount Microplate Scintillation Counter; PerkinElmer).

### miRNA and mRNA analysis by qRT-PCR

RNA from FACS-purified CD44^lo^ naïve CD5^lo^ and CD5^hi^ or CD44^hi^ MP CD8^+^ T cells (∼2–3 × 10^6^ and ∼0.5–1 × 10^6^ cells for naïve and memory subsets, respectively, isolated from pooled B6 LN cells; *n*=2–3 mice) was extracted by using the TRIzol reagent (Life Technologies), followed by column purification using the RNeasy Mini kit (Qiagen) and reversely transcribed with the QuantiTect Reverse Transcription kit (Qiagen) or SuperScript III Reverse transcription (Invitrogen) according to the manufacturers' instructions. TaqMan Gene Expression Assays in combination with the Universal PCR Master Mix and the ABI-Prism 7,900 system (all from Applied Biosystems) were used for quantification of the following housekeeping genes and the genes of interest (all ordered from inventory primers provided by Applied Biosystems): *Dusp5* (Mm01266106_m1), *Dusp6* (Mm00518185_m1), *Ptpn6* (Mm00469153_m1), *Ptpn11* (Mm00448434_m1), *Ptpn22* (Mm00501246_m1), *Gapdh* (Mm99999915_g1) and *Hprt* (Mm01545399_m1). Target gene quantification was normalized to housekeeping gene expression and the data were calculated as the *C*_T_ of target genes normalized to the *C*_T_ of housekeeping gene of each sample. Analyses for miRNA-181a were performed by TaqMan real-time qRT-PCR assay kit (has-miR-181a; Applied Biosystems) on the ABI-Prism 7900 system (Applied Biosystems) and based on the manufacturer's technical recommendation, snoRNA202 was used as the internal control. In each set, the test sample was expressed as relative expression.

### Statistics

An unpaired two-tailed Student's *t*-test was performed to test statistical significance. Differences in mean values were considered statistically significant at a *P* value of <0.05.

### Data availability

All data which support the findings of this study are available within the article (as figure data or Supplementary Information Files) and from the corresponding author upon request.

## Additional information

**How to cite this article:** Cho, J.-H. *et al.* CD45-mediated control of TCR tuning in naïve and memory CD8^+^ T cells. *Nat. Commun.* **7,** 13373 doi: 10.1038/ncomms13373 (2016).

**Publisher's note:** Springer Nature remains neutral with regard to jurisdictional claims in published maps and institutional affiliations.

## Supplementary Material

Supplementary Information Supplementary Figures 1-9.
