# Supplementary material for: CD45-mediated control of TCR tuning in naïve and memory CD8+ T cells
Source: Nat Commun. 2016 Nov 14;7:13373. doi: 10.1038/ncomms13373 (PMC5114568; doi:10.1038/ncomms13373)
Supplement: Supplementary Information — Supplementary Figures 1-9. [file ncomms13373-s1.pdf]

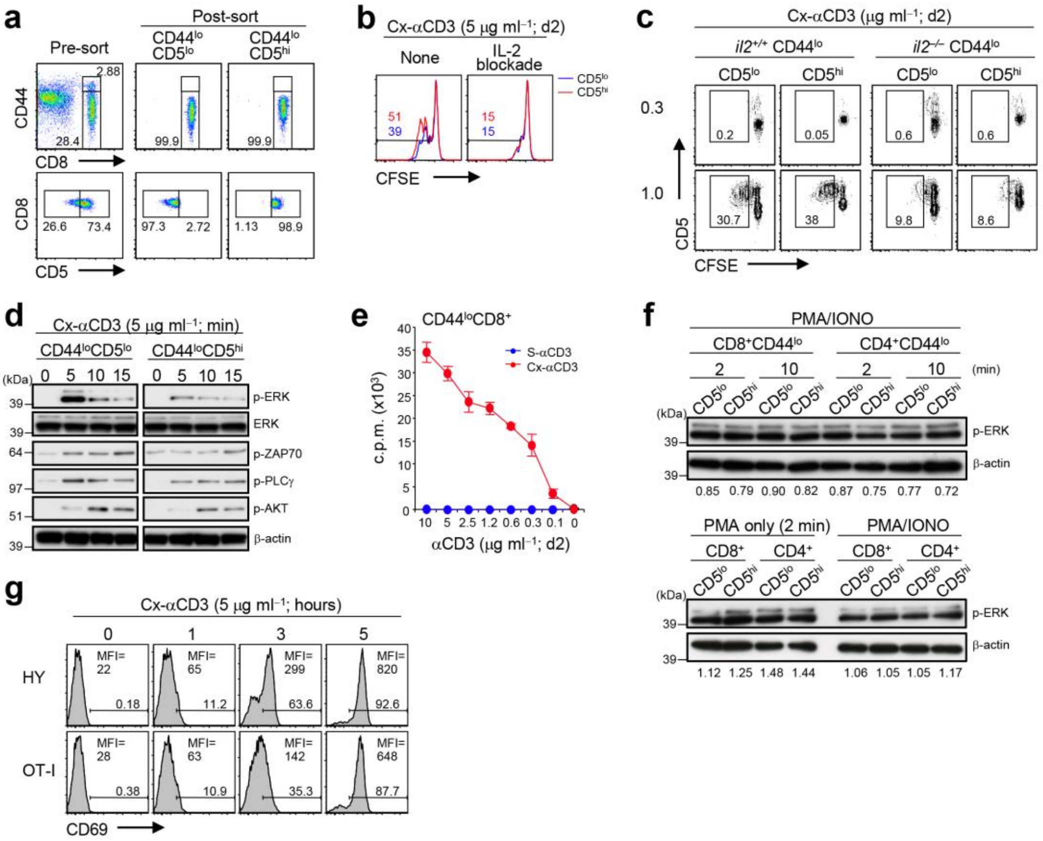

Supplementary Figure 1. IL-2 and TCR responsiveness of $CD5^{lo}$ and $CD5^{hi}$ naïve $CD8^+$ T cells. (a) Flow cytometry for CD44 and CD5 expression in gated $CD8^+$ T cells from B6 LN cells showing purity of each gated population before and after cell sorting. (**b)** Proliferation of CFSE-labeled $CD5^{lo}$ and $CD5^{hi}$ B6 naïve $CD8^+$ T cells after incubation with Cx-αCD3 mAb ± αIL-2 mAb blockade. (**c)** Proliferation of CFSE-labeled $CD5^{lo}$ and $CD5^{hi}$ naïve $CD8^+$ T cells from $il2^{+/+}$ or $il2^{-/-}$ mice after stimulation with the indicated concentrations of Cx-αCD3 mAb. (**d)** Phosphorylation of ERK, ZAP-70, PLCγ, and AKT in $CD5^{lo}$ and $CD5^{hi}$ B6 naïve $CD8^+$ T cells after incubation with Cx-αCD3 mAb. (**e)** Proliferation analysed by [^{3}H]thymidine incorporation of B6 naïve $CD8^+$ T cells after incubation with S-αCD3 or Cx-αCD3 mAb (mean ± s.d.). (**f)** ERK phosphorylation in $CD5^{lo}$ and $CD5^{hi}$ B6 naïve $CD8^+$ and $CD4^+$ T cells after incubation with PMA ± ionomycin. **(g)** Kinetics for CD69 upregulation on HY and OT-I cells after stimulation with Cx-αCD3 mAb (shown with MFI). Data are representative of at least three **(a,b,d,e,g)** and two independent experiments **(c,f)**.

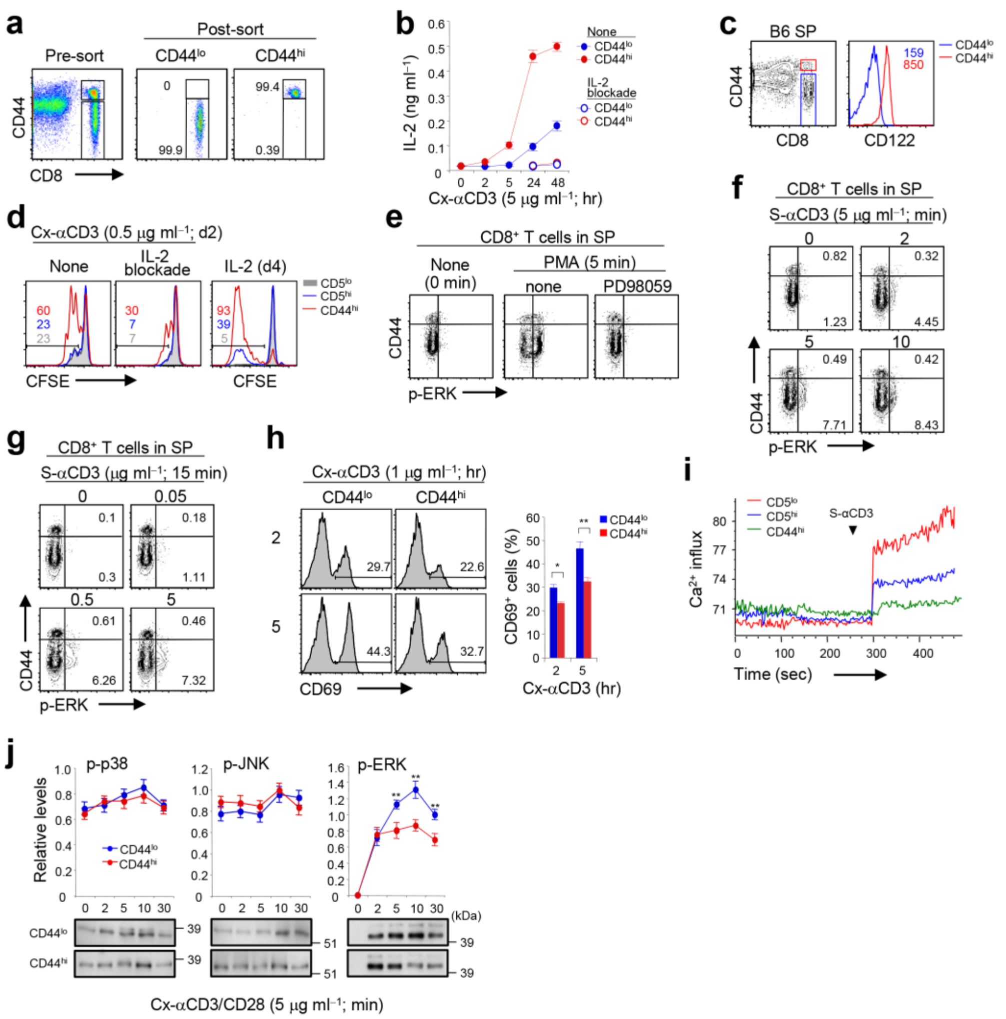

Supplementary Figure 2. TCR responsiveness of CD44^{lo} and CD44^{hi} CD8^{+} T cells. **(a)** Flow cytometry for CD44 expression in CD8^{+} T cells gated from B6 LN cells showing purity of each gated population before and after cell sorting. (**b**) Levels of IL-2 produced by CD44^{lo} and CD44^{hi} CD8^{+} T cells after stimulation with Cx-αCD3 mAb (mean ± s.d.); αIL-2 mAb blockade was added as a background control. (**c**) Flow cytometry for CD122 expression levels on gated CD44^{lo} and CD44^{hi} CD8^{+} T cells from B6 SP cells. (**d**) Proliferation of CFSE-labeled CD44^{lo} CD5^{lo} and CD5^{hi} and CD44^{hi} CD8^{+} T cells after stimulation with Cx-αCD3 mAb ± IL-2 mAb blockade or incubation with IL-2 (10 ng ml^{-1}) without CD3 ligation. **(e-g)** Flow cytometry for detection of ERK phosphorylation by mAb to p-ERK in total B6 SP cells gated on CD8^{+} T cells after incubation with or without either PMA (100 ng ml^{-1}) ± MEK1/2 inhibitor PD98059 (10 μM; **e**) or S-αCD3 mAb (**f**,**g**) for the indicated time points (**f**) or at various concentrations (**g**). **(h)** CD69 upregulation on B6 CD44^{lo} and CD44^{hi} CD8^{+} T cells after incubation with Cx-αCD3 mAb (mean ± s.d.). **(i)** Flow cytometry for Ca^{2+} flux in FACS-purified, Indo-1-loaded B6 CD44^{lo} CD5^{lo} and CD5^{hi} and CD44^{hi} MP CD8^{+} T cells after incubation with S-αCD3 mAb (10 μg ml^{-1}). (**j**) Levels of phosphorylated p38, JNK, and ERK (relative to β-actin; bottom) in CD44^{lo} and CD44^{hi} B6 CD8^{+} T cells after incubation with Cx-αCD3 and -αCD28 mAbs (mean ± s.d.). Data are representative of four **(a,e-g,j)** and three independent experiments **(b-d,h,i)**. Unpaired Student's *t*-test was used for the statistical analysis. *, $P < 0.05$; **, $P < 0.005$.

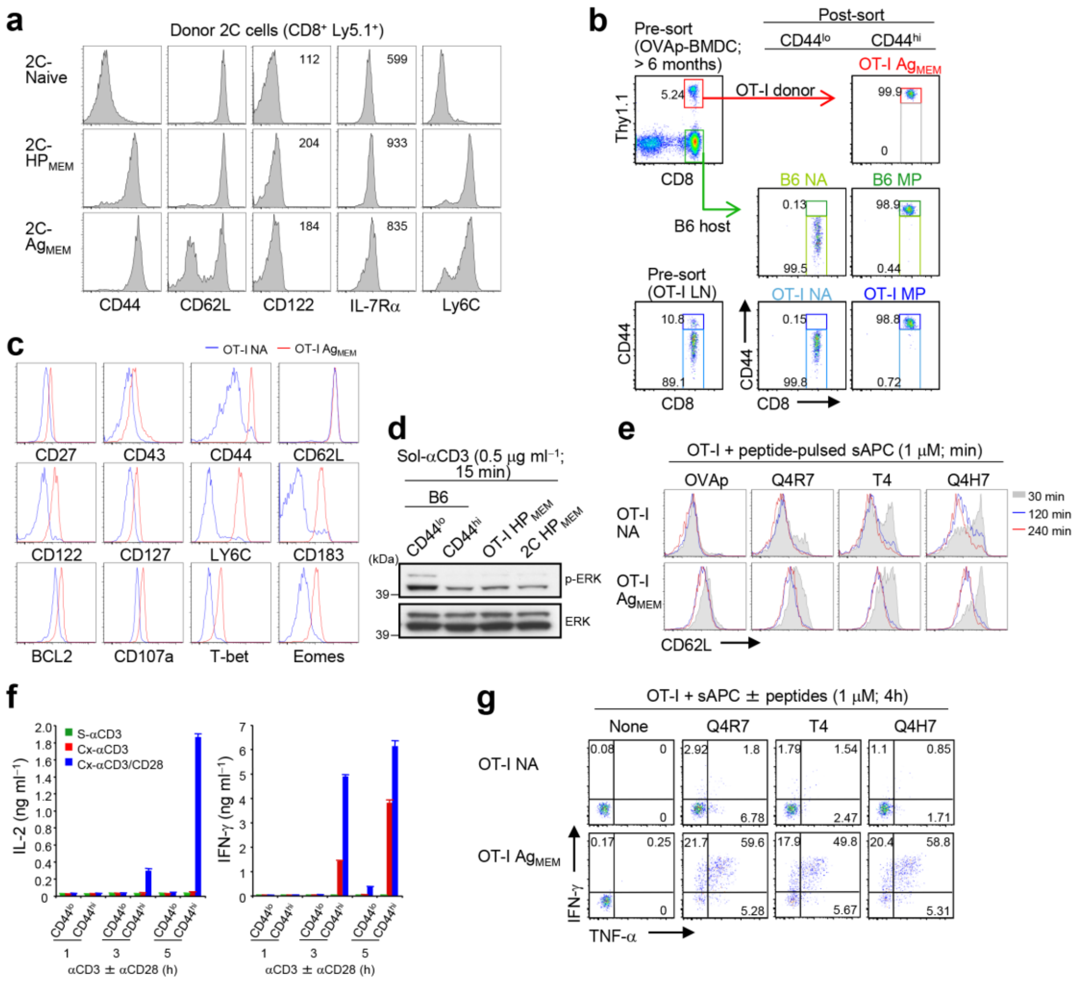

Supplementary Figure 3. Phenotypes, TCR sensitivity and cytokine production of Ag-induced memory CD8$^+$ T cells. (a) Expression of memory cell surface markers on naïve, Ag$_{MEM}$ (cells immunized with specific peptide) and HP$_{MEM}$ (progeny of cells undergoing lymphopenia-induced proliferation) 2C CD8$^+$ T cells obtained as in Fig. 3a. **(b)** Gating strategy for sorting OT-I NA, MP and Ag$_{MEM}$, and B6 NA and MP CD8$^+$ T cells used for Fig. 3b. (**c**) Expression of various memory cell-associated markers compared for OT-I NA versus Ag$_{MEM}$ cells as in (**b**). (**d**) Comparison of ERK phosphorylation in B6 naïve CD44^{lo} cells versus three types of memory CD8$^+$ T cells, B6 CD44^{hi}, OT-I HP$_{MEM}$ and 2C HP$_{MEM}$, after incubation with S-αCD3 mAb. (**e**) Flow cytometry for CD62L downregulation on OT-I naïve (NA) and memory (Ag$_{MEM}$) cells after incubation with sAPC pulsed with indicated peptides as in Fig. 3d. **(f)** Levels of IL-2 and IFN-γ production in CD44^{lo} and CD44^{hi} B6 CD8$^+$ T cells after stimulation with indicated stimuli (mean ± s.d.). **(g)** Flow cytometry for IFN-γ and TNF-α production from OT-I NA versus Ag$_{MEM}$ cells after incubation with sAPC with or without indicated peptides as in Fig. 3f. Data are representative of three **(a-e)** and two independent experiments **(f,g)**.

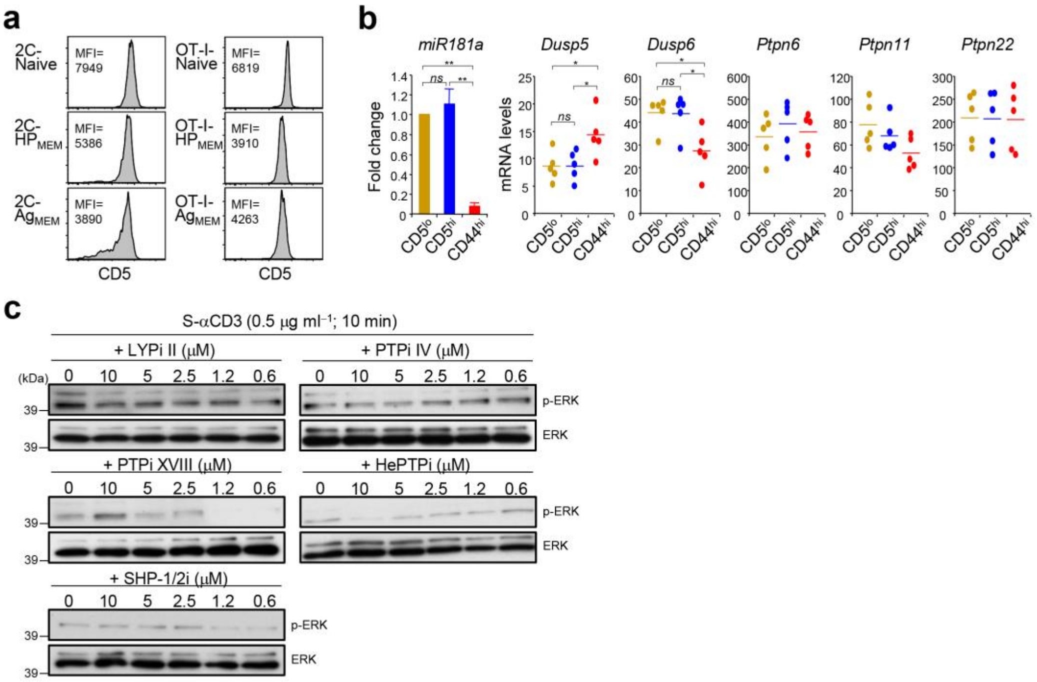

Supplementary Figure 4. Gene expression of various PTPs in CD8$^+$ T cell subsets and influence of PTP inhibitors on TCR sensitivity. (a) Flow cytometry for CD5 expression in 2C or OT-I naïve, Ag_{MEM} and HP_{MEM} CD8$^+$ T cells as in Supplementary Fig. 3. **(b)** Levels of miR181a (mean ± s.d.) and mRNA for the indicated phosphatases in B6 CD44^{lo} CD5^{lo} and CD5^{hi} and CD44^{hi} CD8$^+$ T cells. **(c)** ERK phosphorylation in CD44^{lo} B6 naïve CD8$^+$ T cells after incubation with S-αCD3 mAb ± titrated concentrations of the indicated inhibitors; their specificity for a variety of phosphatases is described in Methods section. Data are representative of three **(a,c)** and five independent experiments **(b)**. Unpaired Student's *t*-test was used for the statistical analysis. *, $P < 0.05$; **, $P < 0.0005$; *ns,* not significant.

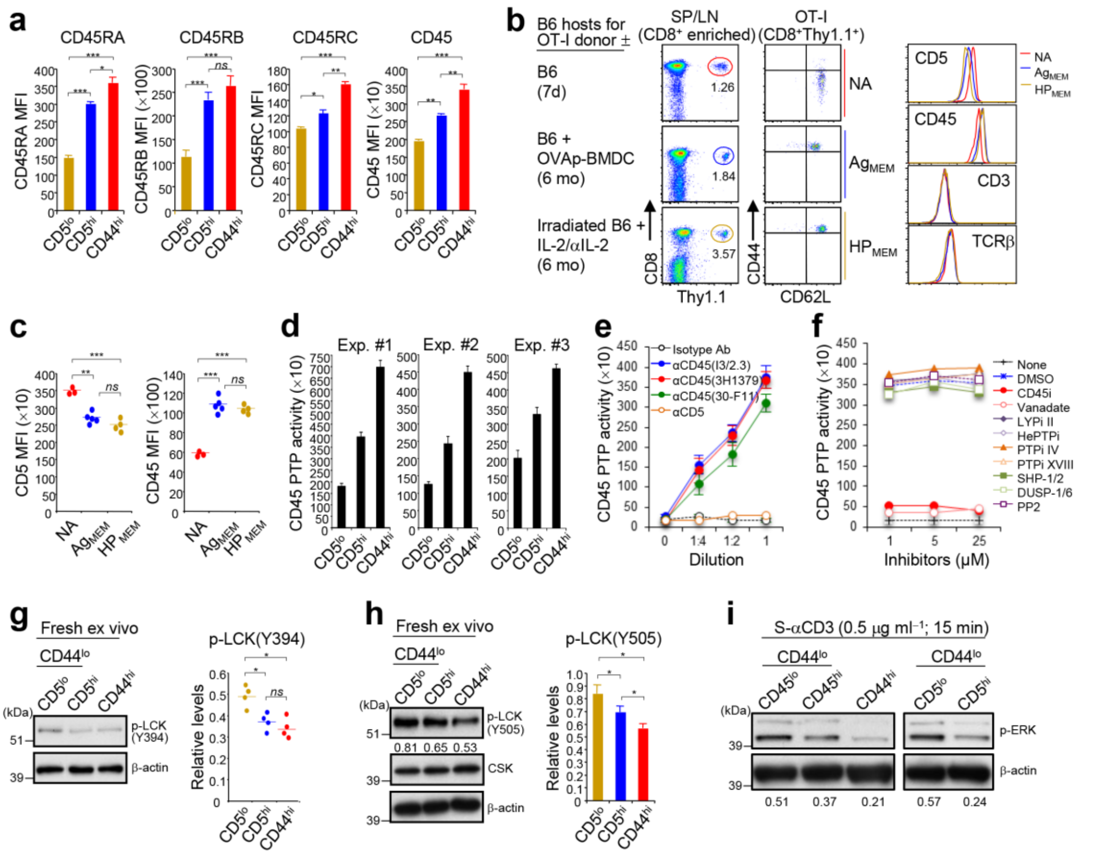

Supplementary Figure 5. Levels of CD45, its PTP activity, and basal p-LCK in CD8$^+$ T cell subsets and effect of CD45 density on TCR sensitivity. (a) Levels of CD45 isoforms and pan-CD45 in B6 CD44^{lo} CD5^{lo} and CD5^{hi} and CD44^{hi} CD8$^+$ T cells (mean ± s.d.; *n* = 8). (**b,c)** Expression of CD5, CD45, CD3 and TCRβ in OT-I NA (*n* = 3), Ag$_{MEM}$ (*n* = 5) and HP$_{MEM}$ CD8$^+$ T cells (*n* = 4; right histogram panel; **b**) gated from the relevant recipient mice indicated (left two panels; **b**) and MFI levels of CD5 and CD45 (**c**); Ag$_{MEM}$ cells were prepared 6 months after priming naïve OT-1 cells by immunization with OVAp-pulsed bone marrow-derived dendritic cells (OVAp-BMDC); HP$_{MEM}$ were prepared by expanding naïve OT-1 cells in irradiated hosts given IL-2/αIL-2mAb complexes. (**d**) *In vitro* PTP activity of CD45 immunoprecipitated by anti-CD45 mAb (3H1379) with whole cell lysates from equal numbers of CD44^{lo} naïve CD5^{lo} and CD5^{hi} cells and CD44^{hi} MP CD8$^+$ T cells. (**e,f**) *In vitro* PTP activity of CD45 immunoprecipitated from cell lysates of naïve CD8$^+$ T cells with three different anti-CD45 mAbs (three clones: I3/2.3, 3H1379, and 30-F11) or as a control by anti-CD5 mAb and isotype mAb without (**e**) or with indicated various PTP inhibitors (**f**). (**g,h**) Levels of basal p-Y394-LCK (**g**) and p-Y505-LCK (**h**) in freshly isolated B6 CD44^{lo} CD5^{lo} and CD5^{hi} and CD44^{hi} CD8$^+$ T cells (**h**; mean ± s.d.). **(i)** Levels of phosphorylated ERK in CD44^{lo} CD45^{lo} versus CD45^{hi} naïve cells and CD5^{lo} versus CD5^{hi} naïve cells and CD44^{hi} MP CD8$^+$ T cells after incubation with S-αCD3 mAb. Data are representative of two **(a-c)**, three **(d-f,i)** and at least four independent experiments **(g,h)**. Unpaired Student's *t*-test was used for the statistical analysis. *, $P < 0.05$; **, $P < 0.0005$; ***, $P < 0.00005$; *ns*, not significant.

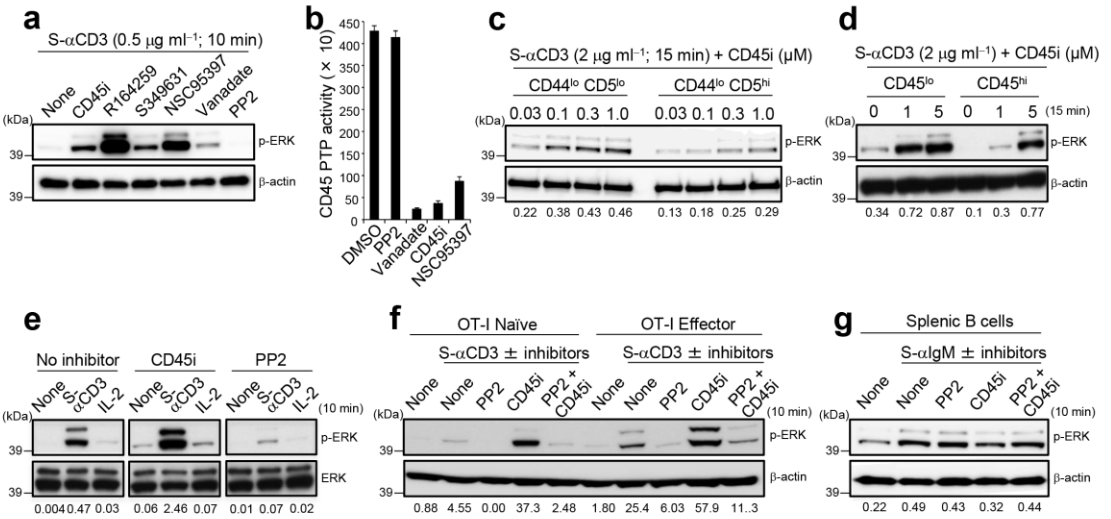

Supplementary Figure 6. Effect of CD45 inhibition on TCR signalling with CD3 ligation and specificity of CD45 inhibitor. (a) ERK phosphorylation in B6 naïve $CD8^+$ T cells after incubation with S-αCD3 mAb ± various inhibitors indicated (CD45i, R164259, S349631, and NSC95397 specific for CD45; vanadate specific for multiple PTPs including CD45; PP2 specific for LCK). **(b)** *In vitro* PTP activity of CD45 immunoprecipitated from whole cell lysates of naïve $CD8^+$ T cells with anti-CD45 mAb (3H1379) without (DMSO) or with indicated PTP inhibitors as in **(a)** (mean ± s.d.). **(c,d)** ERK phosphorylation in $CD44^{lo}$ $CD5^{lo}$ and $CD5^{hi}$ **(c)** and in $CD44^{lo}$ $CD45^{lo}$ and $CD45^{hi}$ B6 naïve $CD8^+$ T cells **(d)** after incubation with S-αCD3 mAb ± CD45i. **(e)** ERK phosphorylation in $CD44^{lo}$ B6 $CD8^+$ T cells after incubation with or without S-αCD3 mAb (0.5 μg ml^{-1}) or IL-2 (1 μg ml^{-1}) ± CD45i (5 μM) or PP2 (2 μM). **(f)** ERK phosphorylation in OT-I naïve and pre-activated (with Cx-αCD3/αCD28 for 2 days) $CD8^+$ T cells after incubation with S-αCD3 mAb (2 μg ml^{-1}) ± PP2 (2 μM), CD45i (5 μM), or both. **(g)** ERK phosphorylation in B6 splenic mature B cells after incubation with anti-mouse IgM $F(ab')_2$ (10 μg ml^{-1}) ± PP2 (2 μM), CD45i (5 μM), or both. Data are representative of three **(a,b,e-g)** and two independent experiments **(c,d)**.

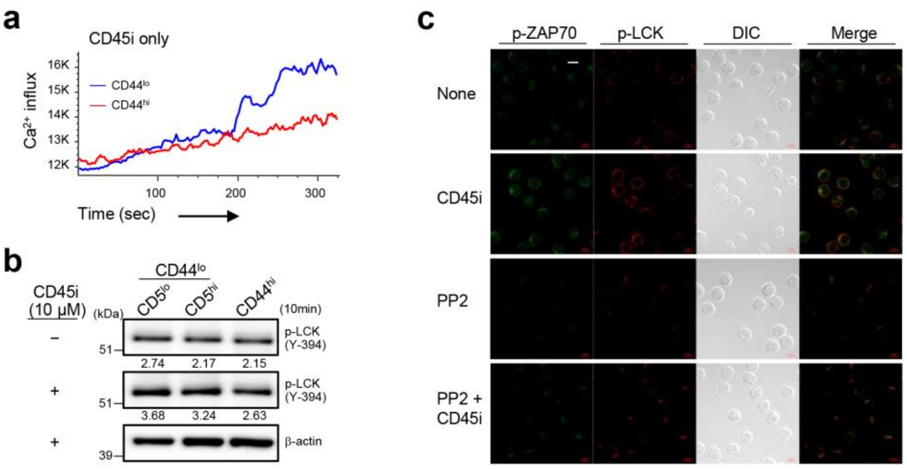

Supplementary Figure 7. Effect of CD45 inhibition on basal tonic TCR signalling. **(a)** Ca^{2+} flux in $CD44^{lo}$ and $CD44^{hi}$ $CD8^{+}$ T cells gated from Indo-1-loaded B6 LN cells in response to CD45i only (2 μM). (**b**) Immunoblotting for p-Y394-LCK in freshly isolated $CD44^{lo}$ naïve $CD5^{lo}$ and $CD5^{hi}$ cells and $CD44^{hi}$ MP $CD8^{+}$ T cells at 15 min after incubation with or without CD45i (10 μM). **(c)** Confocal staining for p-ZAP70 (Y319) and p-LCK (Y394) in freshly isolated naïve $CD8^{+}$ T cells at 15 min after incubation with or without PP2 (2 μM), CD45i (5 μM), or both (original magnification ×63; ×1.4 zoom; scale bars, 5 μm). Data are representative of three **(a)** and two independent experiments **(b,c)**.

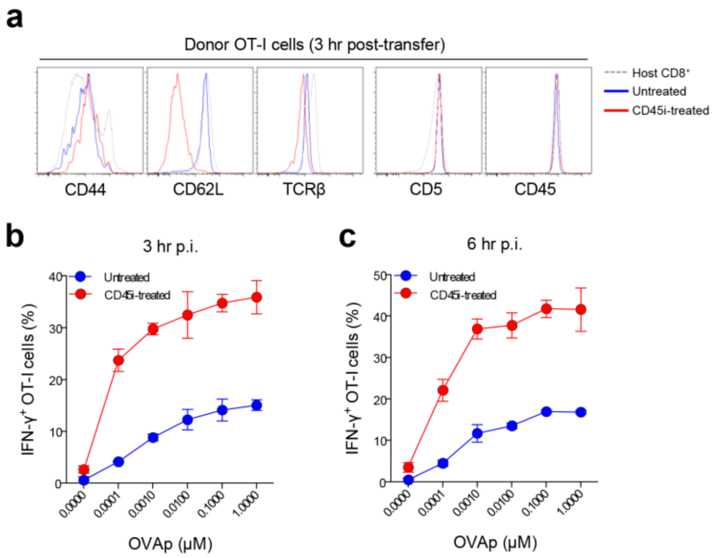

Supplementary Figure 8. Effect of CD45 inhibition on TCR sensitivity to peptide antigen *in vivo*. (a) Expression of various surface markers, CD44, CD62L, TCRβ, CD5 and CD45, on either untreated or CD45i-treated OT-I donor $CD8^+$ T cells; cells were i.v. injected and harvested as in Fig. 8, at 3 hr after adoptive transfer into B6 mice that had been preinjected i.p. with OVAp plus polyI:C. **(b,c)** Spleen cells harvested at 3 **(b)** and 6 hr **(c)** after transfer as in Fig. 8 were cultured *in vitro* with indicated various concentrations of OVAp for 5 hr and analysed for intracellular IFN-γ production by flow cytometry. Data are representative of two independent experiments **(a-c)**.

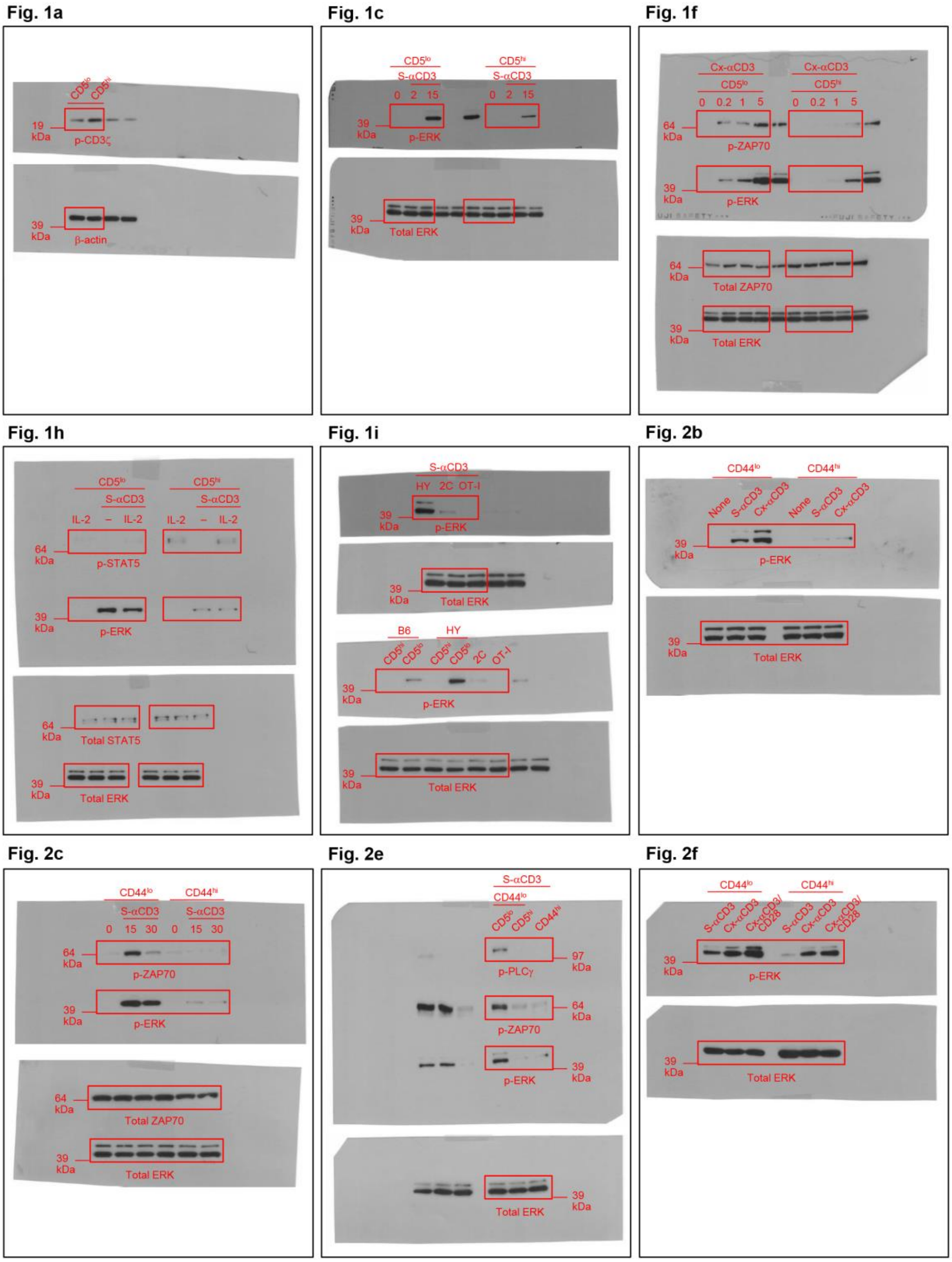

Supplementary Figure 9. Uncropped images of immunoblot. Red boxes show cropped regions.

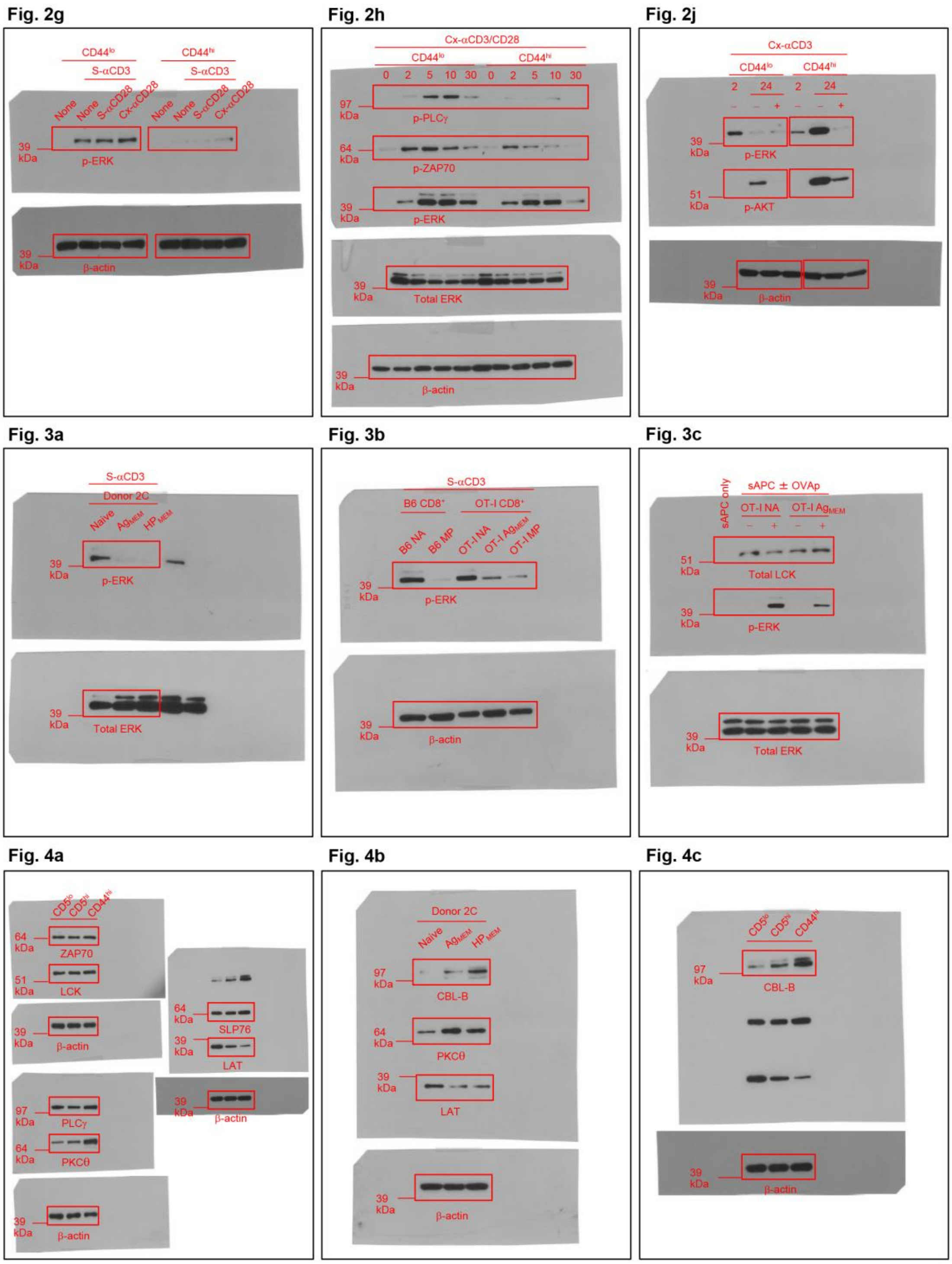

Supplementary Figure 9. Continued.

Fig. 4d

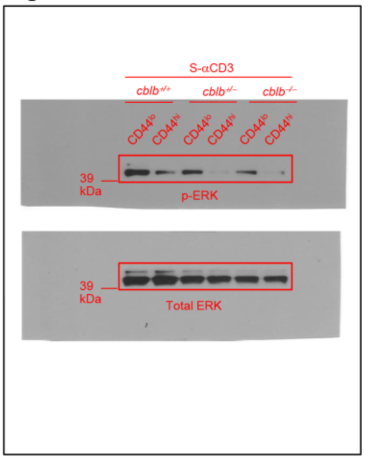

Fig. 4e

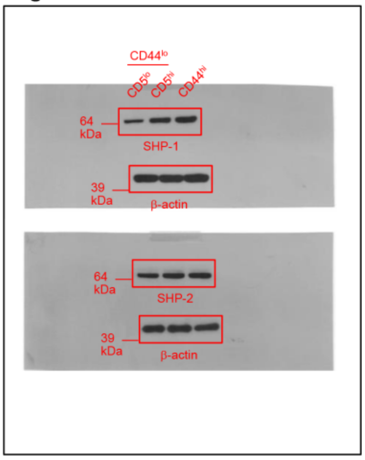

Fig. 4f

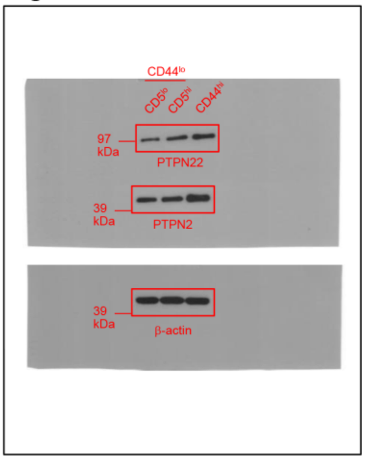

Fig. 4g

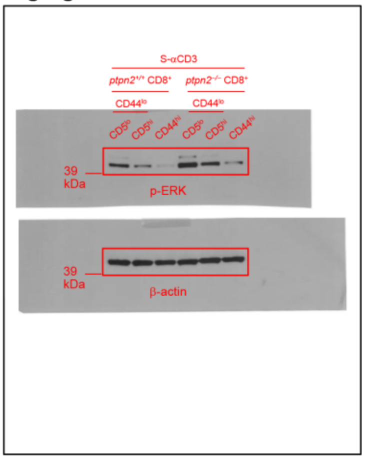

Fig. 4h

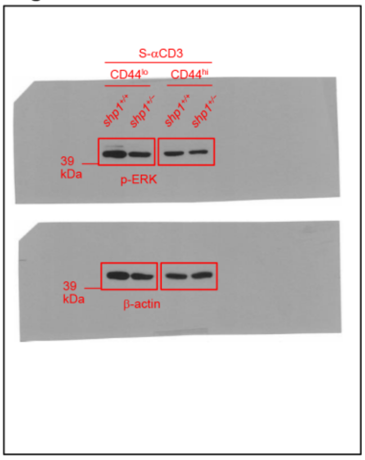

Fig. 5g

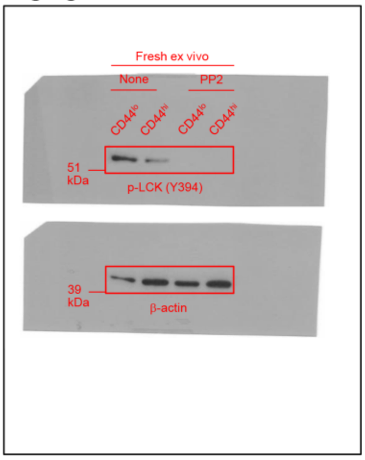

Fig. 5i

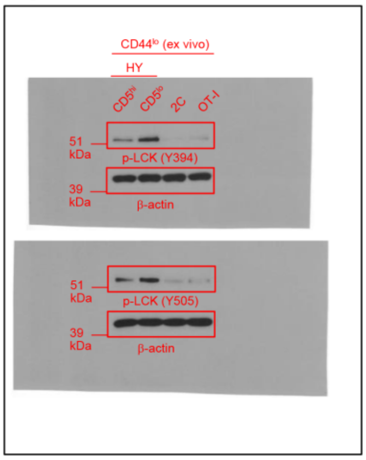

Fig. 5j

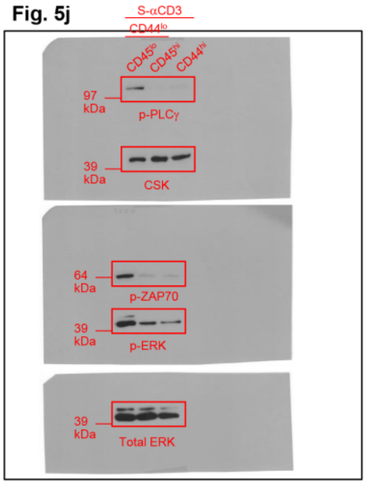

Fig. 6a

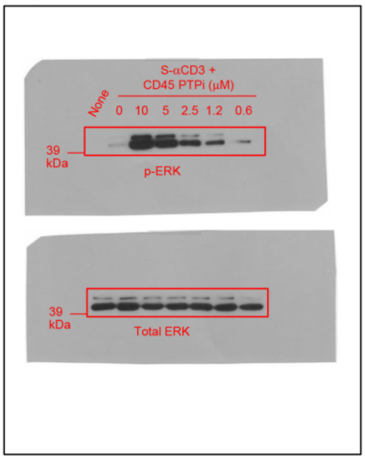

Supplementary Figure 9. Continued.

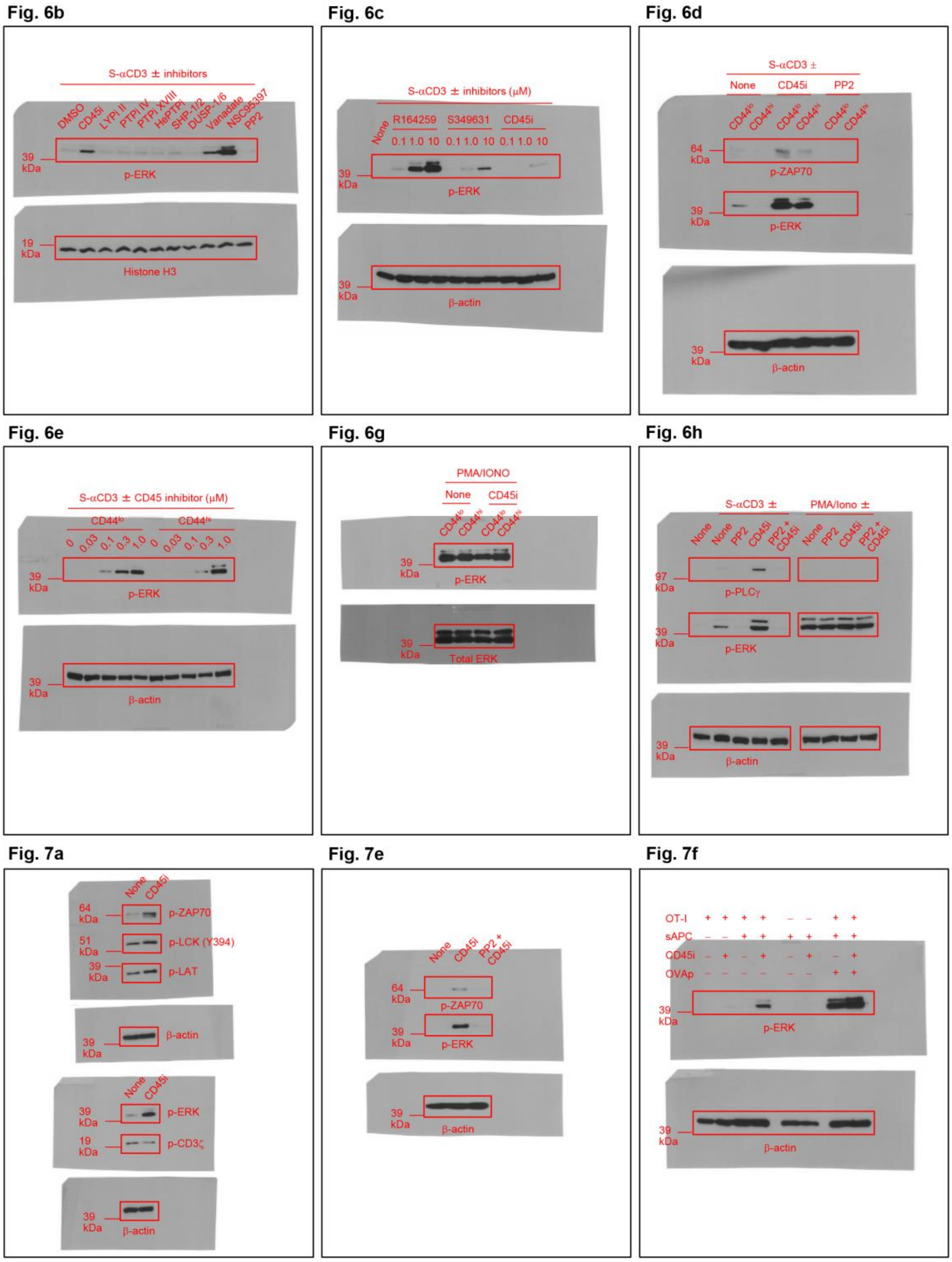

Supplementary Figure 9. Continued.

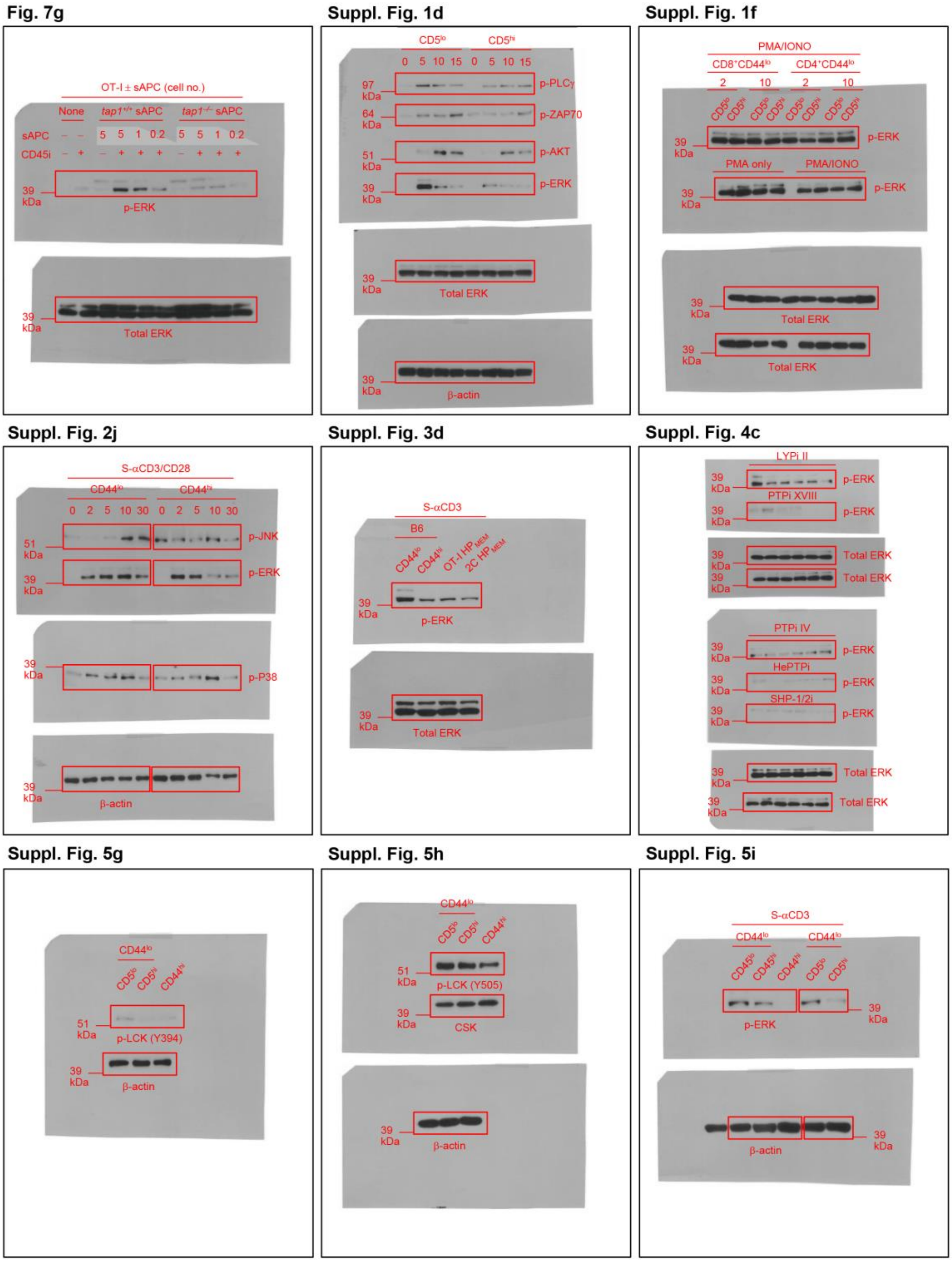

Supplementary Figure 9. Continued.

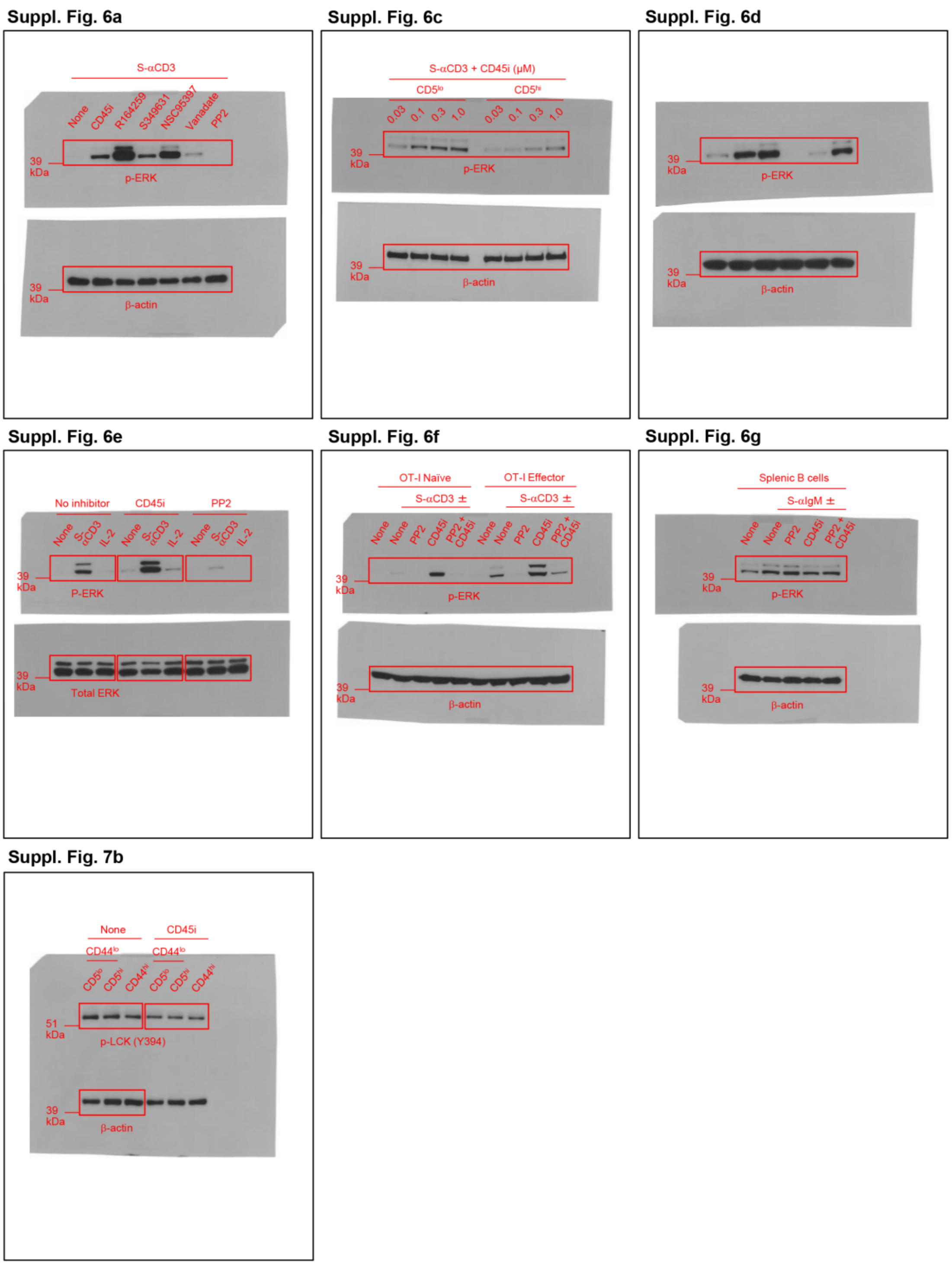

Supplementary Figure 9. Continued.
